# Cellular commitment in the developing cerebellum

**DOI:** 10.3389/fncel.2014.00450

**Published:** 2015-01-12

**Authors:** Hassan Marzban, Marc R. Del Bigio, Javad Alizadeh, Saeid Ghavami, Robby M. Zachariah, Mojgan Rastegar

**Affiliations:** ^1^Department of Human Anatomy and Cell Science, University of ManitobaWinnipeg, MB, Canada; ^2^Department of Pathology, University of ManitobaWinnipeg, MB, Canada; ^3^Department of Biochemistry and Medical Genetics, University of ManitobaWinnipeg, MB, Canada; ^4^Regenerative Medicine Program, University of ManitobaWinnipeg, MB, Canada

**Keywords:** cerebellum structure, apoptosis, autophagy, brain development, epigenetics, DNA methylation, aging

## Abstract

The mammalian cerebellum is located in the posterior cranial fossa and is critical for motor coordination and non-motor functions including cognitive and emotional processes. The anatomical structure of cerebellum is distinct with a three-layered cortex. During development, neurogenesis and fate decisions of cerebellar primordium cells are orchestrated through tightly controlled molecular events involving multiple genetic pathways. In this review, we will highlight the anatomical structure of human and mouse cerebellum, the cellular composition of developing cerebellum, and the underlying gene expression programs involved in cell fate commitments in the cerebellum. A critical evaluation of the cell death literature suggests that apoptosis occurs in ~5% of cerebellar cells, most shortly after mitosis. Apoptosis and cellular autophagy likely play significant roles in cerebellar development, we provide a comprehensive discussion of their role in cerebellar development and organization. We also address the possible function of unfolded protein response in regulation of cerebellar neurogenesis. We discuss recent advancements in understanding the epigenetic signature of cerebellar compartments and possible connections between DNA methylation, microRNAs and cerebellar neurodegeneration. Finally, we discuss genetic diseases associated with cerebellar dysfunction and their role in the aging cerebellum.

## Introduction

Microscopic anatomy of the cerebellum was described in detail at the end of the 19th century by Ramon y Cajal and has attracted the attention of many researchers over the last century, and yet many questions remain unanswered. The role of cerebellum in motor coordination is well studied (Ito, 1984; Glickstein, 1993; Schmahmann, 1997; Glickstein et al., 2009). Increasing evidence shows that the cerebellum also plays a significant role in cognitive functions such as attention, language, emotional behavior, sleep, and even non-somatic visceral responses (Leiner et al., 1991; Wiser et al., 1998; Schmahmann and Caplan, 2006). This review will focus on development of the cerebellum and especially the factors that dictate the generation, migration, and differentiation of neurons. A detailed review of function, physiology, circuitry (White and Sillitoe, 2013a), and neurochemistry (Kwong et al., 2000) is beyond the scope of this paper.

The mammalian cerebellum is characterized by a midline vermis flanked by hemispheres on each side. Folds and fissures divide the cerebellum into lobes, lobules, and folia. Mammalian and avian cerebellum is conventionally divided into 3 lobes that are further subdivided into 10 lobules (I–X) (Larsell, 1970; Sotelo and Wassef, 1991; Voogd and Glickstein, 1998; Glickstein et al., 2009). The cerebellum contains relatively few cell types that are aggregated in the cerebellar gray matter including the cerebellar cortex and cerebellar nuclei. The cerebellar cortex is formed by three layers, whose neuronal components include stellate and basket cells in the molecular layer, Purkinje and candelabrum cells in the Purkinje layer, and granule cells, Golgi cells, unipolar brush cells, and Lugaro cells in the granular layer. Neurons of the cerebellar nuclei are located close to the roof of the fourth ventricle deep within the cerebellar white matter. The cerebellar nuclei along with some vestibular nuclei constitute the sole output of the cerebellum (Ito, 1984; De Zeeuw and Berrebi, 1995; Voogd et al., 1996).

Cerebellar neurons can be classified into inhibitory gamma-butyric acid (GABAergic) and excitatory glutamatergic neurons (Hoshino, 2006b; Carletti and Rossi, 2008). Purkinje cells, which are GABAergic, are the principal neurons of the cerebellar cortex with an elaborate dendritic arborization that extends into the molecular layer. Purkinje cells, the sole output of cerebellar cortex, project to the cerebellar nuclei neurons (Leto and Rossi, 2012; Mordel et al., 2013; Steuber and Jaeger, 2013). Candelabrum cells are also GABAergic; they are uniformly distributed throughout the entire cerebellar cortex. The small somata are roughly pyriform and vertically located between the Purkinje cell somata (Lainé and Axelrad, 1994; Ambrosi et al., 2007; Carletti and Rossi, 2008). Other cerebellar cortex GABAergic interneurons include basket and stellate cells in the molecular layer, and Golgi and Lugaro cells located in the granular layer (Leto et al., 2008; Leto and Rossi, 2012; Castejón, 2013).

Glutamatergic neurons include the granule cells, unipolar brush cells, and excitatory projection neurons (large neurons in the cerebellar nuclei) (Lainé and Axelrad, 1994; Voogd and Glickstein, 1998; Cotterill, 2001; Sillitoe and Joyner, 2007). The cerebellar granule cells are the smallest and most abundant neurons in the vertebrate brain, representing about 80% of all neurons in the human brain (Azevedo et al., 2009). The small cell bodies are packed into the granular layer with about 4–5 dendrites that receive cerebellar afferent input (Goldowitz et al., 1997; Kalinichenko and Okhotin, 2005; Chédotal, 2010). Unipolar brush cells are small glutamatergic neurons residing in the granular layer of the cerebellar cortex; they amplify signals from afferent fibers (Kalinichenko and Okhotin, 2005; Mugnaini et al., 2011).

The cerebellar nuclei are comprised of four major subdivisions: (A) the medial (fastigial), which is subdivided further into caudomedial, middle, and dorsolateral (rostrolateral) nuclei; (B) anterior and posterior interposed nuclei; and (C) lateral (dentate) nuclei (De Zeeuw and Berrebi, 1995; Manto et al., 2013). The medial nuclei generally communicate with the vermis, the interposed nuclei with the paravermis, and the lateral nuclei with the hemispheres (Voogd and Glickstein, 1998). Cerebellar nuclei are composed of several neuronal types: excitatory glutamatergic neurons, which project to different parts of the brain, inhibitory GABAergic neurons that terminate in the inferior olive (Ruigrok, 1997), and inhibitory GABAergic and glycinergic interneurons (Uusisaari et al., 2007; Uusisaari and Knöpfel, 2012).

The cerebellum receives two major and one minor types of afferent input. Mossy fibers constitute the majority of afferent fibers in the adult cerebellum. Arising from multiple sources in the central nervous system, they project to the Purkinje cells through granule cells/parallel fibers (Valle et al., 2001; Voogd et al., 2003; Voogd, 2011). Climbing fibers are exclusively derived from the inferior olivary complex; they synapse on the dendrites of Purkinje cells (Marani and Voogd, 1979; Campbell and Armstrong, 1983). A third set of afferents called neuromodulatory cerebellar afferents terminate in all three layers of the cerebellar cortex (Jaarsma et al., 1997; Schweighofer et al., 2004; Manto et al., 2013). All afferents to the cerebellum also send a direct branch to the cerebellar nuclei; these nuclei neurons also receive the Purkinje cell input that are essential in monitoring the whole cerebellar output (Marzban et al., 2010; Hashimoto and Hibi, 2012).

The fundamental architecture of the cerebellum is organized into four transverse zones based on gene expression and afferent fiber termination; the anterior zone (AZ: corresponding approximately to lobules I–V in mice), the central zone (CZ: lobules VI–VII), which can be further subdivided into anterior (CZa) and posterior (CZp) components (Marzban et al., 2008; Sawada et al., 2008), the posterior zone (PZ: lobules VIII–IX), and the nodular zone (NZ: lobules IX–X; Ji and Hawkes, 1994; Marzban et al., 2003, 2004, 2012; Sugihara and Quy, 2007; Marzban and Hawkes, 2011; Bailey et al., 2014). The boundaries of these zones do not align absolutely with the lobe and lobule divisions, but provide a more functionally relevant way of dividing the cerebellum (Marzban et al., 2011, 2014). The cerebellum contains the most elaborately patterned circuit of all the central nervous system structures, which may be essential for organizing the large number of functional and topographic zonal circuits (Reeber et al., 2013; White and Sillitoe, 2013b). Studies on gene expression patterns in cerebellar nuclei neurons have also revealed molecular heterogeneity that may mirror the molecular complexity of the cerebellar cortex (Chung et al., 2009).

The principal cerebellar cytostructure is set during early development and precedes the process of neurogenesis and axonogenesis during which cerebellar circuits and functions are established. Here, we will first describe cerebellar development in humans, which begins during embryonic development and continues into early childhood. We will then review, lower mammalian cerebellar development, highlighting the molecular and genetic aspects of the cerebellar primordium, germinal zones, and neurogenesis that cannot be directly studied in humans. Finally, we will discuss the latest advancements in the study of genetic, epigenetic and molecular signaling pathways in cerebellum development.

## Embryonic and early fetal development of the human cerebellum

Detailed morphologic descriptions of human cerebellum development in the embryonic period come mainly from the work of Müller and O’Rahilly, although some information had been published earlier. At Carnegie stage 13 (28 days post fertilization) the cerebellar plate begins as a bulge on the dorsal aspect of the rhombencephalon (alar plate of the rhombomere 1), dorsolateral to the sulcus limitans on the floor of the fourth ventricle (Müller and O’Rahilly, 1988a). At stage 14 (32 days) the cerebellar primordium expands rapidly with a thick ventricular layer comprised of radially oriented cells, a less cellular intermediate layer, and a marginal layer (Bogaert and Belpaire, 1977). At stage 15 (36 days) the two sides of the cerebellar primordium are not yet in contact and are spanned by the thin rhombencephalic roof plate (Müller and O’Rahilly, 1988b). The rhombic lip, a dorsolateral proliferative area with mitotic activity, is established at stage 16 (40 days) (Müller and O’Rahilly, 1989). Neuron progenitors begin migrating from the ventricular zone (VZ) at stages 18 and 19 (44–48 days) (Müller and O’Rahilly, 1990a). By stage 20 (52 days) cells that will form the dentate nucleus migrate radially from the VZ and rostromedially from the rhombic lip. At stage 20 the superior cerebellar peduncle appears. At stage 21 the dorsal projection of the cerebellar plate thickens. The cerebellar commissures (rudiments of the vermis) are first apparent at stage 22. At stage 23 (approximately 57 days/8 weeks; the end of the embryonic period) the external germinal zone (EGZ; usually referred to as the external granular layer in human literature) extends from the rhombic lip onto the dorsum of the cerebellar bulge. The cerebellar plate arches over the brainstem in a U-shape (Müller and O’Rahilly, 1990b).

Cho et al. studied a separate set of embryonic and fetal specimens age at 6–16 weeks gestation (Cho et al., 2011). They reported that the first identifiable cerebellar feature was a thickening on the dorsolateral aspect of the alar plate facing the fourth ventricle at 6 weeks. At the end of embryonic period and beginning of fetal period, from 7–9 weeks, the rhombic lips and alar plates expanded to form the anlages of the cerebellar hemispheres, which began to approximate over the fourth ventricle. By 10 weeks the cerebellar anlages were fused in midline. From 11 to 12 weeks cerebellar posterolateral and primary fissures developed in the vermis and the EGZ became distinct. At 15–16 weeks fissures deepened, more so in the hemispheres, and the dentate nucleus was apparent.

Rakic and Sidman characterized the layers of the cerebellum from 7 to 40 weeks gestation and into adulthood (Rakic and Sidman, 1970). Cell proliferation was restricted to the VZ up to 10 weeks gestation at which time outward radial migration created a 2-layer stage. The EGZ appeared as a distinct layer at 10–11 weeks (3-layer stage), and Purkinje cell plate established by 13 weeks. At 20–21 weeks the hypocellular lamina dissecans became evident deep to the marginal (future molecular) layer, and the 5-layer appearance persisted to 32 weeks. The lamina dissecans disappeared (4-layer stage) at 32 weeks and the EGZ disappeared postnatally (3-layer stage). Similar histologic progression has been reported by others (Milosevic and Zecevic, 1998).

## Fetal and postnatal development of the human cerebellum

During the fetal period, the infratentorial (cerebellum plus brainstem) part of the brain is >5% of the total weight from 14 to 17 weeks, about 5% from 18 to 29 weeks, and exceeds 6.5% by 40 weeks (Guihard-Costa and Larroche, 1990). Between birth and 9 months the cerebellum increases from 5.7 to 10% of the total brain weight. Thereafter the growth rate is the same as brain growth overall and the proportionate weight is constant (Ellis, 1920).

Nowakowska-Kotas et al. studied the lobular morphology of human cerebellum from gestational weeks 15 to 28 (Nowakowska-Kotas et al., 2014). During this interval the flocculonodular lobe volume decreases proportionately from 9 to 6%, the anterior lobe increases from 11 to 22%, and the posterior lobe occupies 80 to 72%. They found a 3.5-fold increase in the exterior cerebellar surface. Taking into account the real surface area along folia, this corresponds to a 30-fold increase in the total surface area (Lemire, 1975).

The vermis begins to develop folia by 13–14 weeks and all of the midline lobules can be identified by 15 weeks. Development of the folia is completed approximately 2 months after term birth (Loeser et al., 1972). From 17 to 29 weeks the relative sizes of the lobules is maintained even as the secondary and tertiary braches of the lobules develop (Kapur et al., 2009).

Developmental morphology of Purkinje neurons has been studied in detail using Golgi impregnation and electron microscopy beginning at 12 weeks gestation. Between 12 and 16 weeks they are small and several rows deep. Between 16–28 weeks they become organized into a single row, enlarge, and develop increasing complex dendritic branches and synapses. Synaptic complexity continues to increase into the first postnatal year (Zecevic and Rakic, 1976). Purkinje neurons rapidly expand in volume between birth and 2 years, and then again around 7–9 years age when adult size is reached (Tsekhmistrenko, 1999). Neurons of the granular layer become progressively more clustered, reaching a plateau at approximately 14–15 years (Tsekhmistrenko, 2001). The prolonged development differs considerably from non-primate species (Zecevic and Rakic, 1976).

Friede documented thickness of the layers of the cerebellum from 24 weeks gestation to 13 months postnatal (Friede, 1973). He reported that the EGZ is fairly stable until approximately 2 months postnatal after which it gradually disappears by 12 months. The molecular layer thickens rapidly between 38 weeks gestation and 1 year postnatal. The Purkinje cell bodies become obvious at about 28 weeks in the vermis and 32 weeks in the hemispheres. Irregular clusters of immature appearing “matrix” cells persist in the deep cerebellar white matter around the cerebellar nuclei until ~4 months, involuting along with the EGZ; the fate of these cells is unclear.

Abrahám et al. studied brains from 24 weeks gestation to 11 months postnatal. They documented layer thickness and cell proliferation (based on Ki67 immunoreactivity) in the EGZ, molecular layer, and granular layer (Abrahám et al., 2001). More than 50% of cells in the EGZ are Ki67 positive from 24 to 34 weeks gestation. The width of the EGZ peaks at 34 weeks and diminishes rapidly between 1 and 9 months postnatal with proliferation negligible after 5–6 months. Width of the molecular layer expanded through the full age range studied. The rate of granular layer expansion is greatest in the vermis and hemispheres from approximately 22 to 32 weeks gestation and is more gradual in the flocculus (Gudovic et al., 1998). During the early fetal period the EGZ is generally a fairly regular layer, but from 25 weeks gestation onward in approximately half of autopsy specimens the EGZ has a regular knobby appearance with clusters of EGZ cells punctuated by penetrating blood vessels (Gelpi et al., 2013). By the third trimester the outer layer of the EGZ (adjacent to the pial surface) is densely packed and contains the proliferating precursor cells. The inner layer of the EGZ is less densely packed and is composed of postmitotic migrating neurons (Haldipur et al., 2011). Gadson (Gadson and Emery, 1976) found that the EGZ disappears between 12 and 18 postnatal months. The cell density of the granular layer reaches adult levels by 2 years age.

## Cell death in the developing human cerebellum

While studying cerebellar cell proliferation, Abrahám et al. also documented dying cells (Abrahám et al., 2001). The percentage of pyknotic nuclei, presumed to be dying cells, was approximately 0.5% in the EGZ from 24 to 34 weeks and between 0.06 and 0.3% to 9 months. In the granular layer there were fewer dying cells, typically <0.1% at all ages studied. Nat et al. studied cell death from 8 to 22 weeks gestation (Nat et al., 2001) using terminal deoxynucleotidyl transferase-mediated dUTP nick end labeling (TUNEL; see limitations of this method discussed below). At 8 weeks the proportion of TUNEL positive cells in the proliferative VZ was 34 ± 7% and in the postmitotic region (i.e., the cerebellar plate) was 18 ± 8%. At 12 weeks, TUNEL positive cells were 20 ± 8% in the VZ and 31 ± 5% in the EGZ. At 15–22 weeks, the granular layer had 5 ± 2% and the Purkinje cell layer 4 ± 1% TUNEL positive cells. At all ages, only about 5% of apoptotic cells were immunoreactive for the cell “suicide” receptor Fas (APO-1/CD95).

Lavezzi et al. (2006) studied brains from 17 weeks gestation to 12 months postnatal using proliferating cell nuclear antigen (PCNA) immunostaining, which yields some information about cell proliferation, and TUNEL. They reported their data semiquantitatively. PCNA positive cells were present in all layers of the cerebellum from 17 to 25 weeks gestation. Labeling remained abundant (>30% of cells) in the EGZ and sparse in the molecular and granular layers to 39 weeks gestation and ceased to be detectable in any layer after 3 months postnatal. TUNEL positive cells were restricted to the EGZ, first apparent sparsely at 26 weeks gestation and abundantly from 4 to 12 months postnatal, by which time the EGZ had disappeared. The authors observed that TUNEL positive cells lacked the morphologic features of dying cells and were negative for BCL2. Simonati and coworkers used TUNEL in fetal brains 12–24 weeks gestation and in three neonatal brains (Simonati et al., 1997, 1999). Scattered TUNEL positive cells were present in the granular layer at all ages but labeled nuclei were not observed in the EGZ, Purkinje cell layer, or in the dentate nucleus. They concluded that mainly glial cells undergo apoptosis in developing cerebellum.

Lossi and coworkers studied cell death in postnatal human cerebellum from term birth to adulthood using TUNEL and T4 DNA ligase methods (Lossi et al., 1998). They used anti-Ki67 to label proliferating cells. From birth to 3 months >30% of cells in the EGZ were Ki67 positive, after which proliferation declined. TUNEL labeling was observed in the EGZ (4–8% of cells), in the granular layer (<2% of cells), and in the white matter (<3% of cells) from birth to 3 months and not thereafter. Seemingly apoptotic cells were often immunoreactive for CPP32/ interleukin-1 beta-converting enzyme but negative for Bcl-2. The authors remarked on the relatively low frequency of cell death in human brains and discussed possible explanations for interspecies differences (e.g., Wood et al., 1993; Krueger et al., 1995). BCL-X, which is related to BCL-2, was reported to be highly expressed in the human fetal cerebellum (Sohma et al., 1996).

In summary, TUNEL labeling in the developing human cerebellum has been documented, but the reported frequency varies considerably. Limitations of the TUNEL method include the lack of specificity for apoptosis and propensity for false positive labeling (Chan et al., 2002; Loo, 2011). Fixative type and delays influence TUNEL positivity (Tamura et al., 2000); fixation can vary considerably in autopsy material. Unfortunately there are no comprehensive studies of cell death developing human cerebellum using other methods. It is not clear to what extent apoptosis and synapse elimination (Hashimoto and Kano, 2013), which has not been studied in humans, are related.

Despite fairly clear morphologic and histologic details about fetal and postnatal cerebellum development in humans, the early embryologic features and the molecular/genetic determinants are not easily studied. Information about early cerebellar development has been acquired from studies of lower animals that have very primitive cerebellum-like structures and from experiments in other mammals, especially rodents.

## Cerebellum and cerebellum-like structures

The basic features of cerebellar architecture are present in primitive species such as hagfish (myxinoids) and lampreys and are called cerebellum-like (cerebelloid) structures (Larsell, 1967). The cerebellum-like structures seem to have parallel fibers and interneurons similar to the molecular layer. Classically, the main component of cerebellum-like structures comprises the medial octavolateral nucleus (MON) and dorsal octavolateral nucleus (DON; Yopak and Montgomery, 2008; Kajiura et al., 2010; Yopak, 2012; Kaslin and Brand, 2013).

Cerebellum-like structures arise from the alar plate of the neural tube (Gao et al., 1996). They are comprised of a layer of principal cells, the exact nature of which is not clear. These cells are probably analogous to the Purkinje cells (Devor, 2000) or neurons of the cerebellar nuclei (Montgomery et al., 2012). It has been shown that the MON receives direct afferent nerves from the mechanosensory receptors of the lateral line system and the DON receives afferents from the electrosensory receptors of the ampullae of Lorenzini (Devor, 2000; Montgomery et al., 2012). In addition to direct projections, the afferents to cerebellum-like structures can be categorized into two major types: parallel fibers and primary afferents. The cell bodies of parallel fibers are called granule cells, which are driven by multiple sources including the spinal cord, brain stem, and cerebrum (Devor, 2000). The primary afferent input in the case of cerebellar-like structures may be analogous to climbing fibers from the inferior olivary nucleus and originate from the octavolateral end organs (Devor, 2000). However, it is also believed that the cerebellar-like structures do not have climbing fiber projections (Montgomery et al., 2012). The similarities and differences of cerebellum and cerebellum-like structures have been reviewed in detail elsewhere (Devor, 2000; Bell, 2002; Montgomery et al., 2012).

Evolutionary appearance of the cerebellum occurred at the juncture between early vertebrates and gnathostomes (jawed vertebrates) (Striedter, 2005). The MON is evident in some hagfish (myxinoids) (Larsell, 1967; Ronan and Northcutt, 1998; Northcutt, 2002), whilst, the two octavolateralis nuclei; i.e., MON and DON are found in lampreys, which lack a true cerebellum (Larsell, 1967; Ronan and Northcutt, 1998; Weigle and Northcutt, 1999; Northcutt, 2002). In addition to the MON and DON, cartilaginous fishes have well defined cerebellum. It has been suggested that the cerebellum arose through a change in the genetic-developmental program, amounting to a duplication of existing structure (e.g., the MON) (Montgomery et al., 2012). Teleost fish lack distinct cerebellar nuclei while lampreys and sharks have at least one cerebellar nucleus (Butler and Hodos, 2005; Kaslin and Brand, 2013). However, because the cerebellar function relies on the cerebellar nuclei neurons as the sole output, it is a matter of debate whether structures lacking cerebellar nuclei should be considered a cerebellum/cerebelloid structure.

It has been suggested that the cerebellum can be considered an example of “subsumption architecture”, in which the new cytoarchitecture and circuits are while maintaining existing fundamental components to improve functionality during evolution (Montgomery et al., 2012; Butts et al., 2014).

## Cerebellar development and neurogenesis

The mouse cerebellar primordium emerges at about embryonic day (E) 7–8 as a neuroepithelial swelling of the rostral lip of the fourth ventricle, which is part of the alar plate of the metencephalon (rhombomere-1/r1) (Goldowitz and Hamre, 1998; Wingate and Hatten, 1999; Wang and Zoghbi, 2001; Lumsden, 2004; Wingate, 2005). The cerebellar primordium develops from a region of neural tube characterized by expression of *Gbx2* and lacking expression of *Otx2* and *Hoxa2* (Butts et al., 2014). Shortly after the cerebellar primordium is formed as a separate simple plate in the dorsal r1, a 90° rotation changes the direction of axes and causes midline fusion (Alvarez Otero et al., 1993; Sgaier et al., 2005). Consequently, the rostral-medial ends of the bilateral primordia form the vermis and the caudal-lateral part becomes the hemisphere of the cerebellum (Louvi et al., 2003). The narrow ring encircling the neural tube between the mesencephalon and rhombencephalon, called the isthmus, forms the anterior boundary of the cerebellar primordium. The isthmus contains the “isthmic organizer”, which is important in development and maintaining the mesencephalon (rostrally) and rhombencephalon (caudally; rhombomere 1) (Itasaki and Nakamura, 1992; Martínez, 2001; Rhinn and Brand, 2001; Wurst and Bally-Cuif, 2001; Partanen, 2007; Crespo-Enriquez et al., 2012). The earliest molecular specification of the isthmic organizer is the interaction between homeodomain transcription factors OTX2 in the rostral epithelium and GBX2 in the caudal domain (Wassarman et al., 1997; Shamim and Mason, 1998; Katahira et al., 2000; Martinez et al., 2013). Important signaling molecules secreted by the isthmic organizer include members of the fibroblast growth factor family such as FGF8 (Heikinheimo et al., 1994; Martinez et al., 2013). *Fgf8* expression is revealed by *in situ* hybridization at E8.5 in mice at the interface of *Otx2* and *Gbx2* positive neuroepithelial cells (Martinez et al., 2013). Similar to GBX2, which induces cerebellar primordium formation in r1 by inhibition of *Otx2*, FGF8 signaling appears to act also by inhibiting *Otx2* in the r1. It was shown that FGF8 is required for development of the vermis; reduction of FGF8 is associated with expansion of *Otx2* expression in alar plate of r1 (Butts et al., 2014). *Otx2* and *Gbx2* expression are fundamental for positioning the isthmic organizer and for the establishment of molecular interactions of FGF8, EN1, EN2, WNT1, PAX2, Iroquas (IRXS), Sonic Hedgehog (SHH), and transforming growth factor (TGF)-β family member expression (Danielian and McMahon, 1996; Hidalgo-Sánchez et al., 2005; Blaess et al., 2006; Vieira et al., 2010; Martinez et al., 2013). Temporo-spatial patterns of such gene expressions are necessary for the normal development of the cerebellum particularly in the anterior region. For instance, *En1* and *En2* expression extends over the rostral cerebellar territory from the mesencephalon at ~E9 in mice. Some mouse strains with *En1* and *En2* mutations (meander tail, leaner) have anterior cerebellar anomalies (Ross et al., 1990; McMahon et al., 1992). Depending on the developmental stage, the “isthmic organizer” controls a variety of processes such as cell survival, cell identity, neural precursor proliferation, neuronal differentiation, and axon guidance (Millet et al., 1996; Martínez, 2001; Martinez et al., 2013). The caudal boundary is defined by the roof plate of the fourth ventricle (Lee et al., 2000; Millonig et al., 2000; Chizhikov et al., 2006a), where *Hox2* expression is present in r1 but not r2 (Wurst and Bally-Cuif, 2001). The roof plate is a source of bone morphogenetic protein (BMP) family members that can induce expression of *Math1*, an important factor in cerebellar primordium development (Alder et al., 1999).

Early cerebellar primordium neuronal populations have been mapped based on gene expression. Expression patterns of specific transcription factors that define four distinct cellular domains during early development (~E10–E12) are summarized in Table 1 (Chizhikov et al., 2006a; Zordan et al., 2008).

**Table 1:** **Neuronal populations within the cerebellar primordium**.

Domain	Genes	Phenotypes	Neurons
C1	*Math1* *Lmx1a*	Glutamatergic	Large neurons of the cerebellar nuclei, granule cells, unipolar brush cell
C2	*Lhx1/5* *Ptf1a*	GABAergic	Purkinje cells interneurons
C3	*Lmx1a* *Tbr1*	Glutamatergic	Large neurons of the cerebellar nuclei
C4	*Lhx1/5*	?	?

### Germinal zones of the developing cerebellum

**Figure 1:**
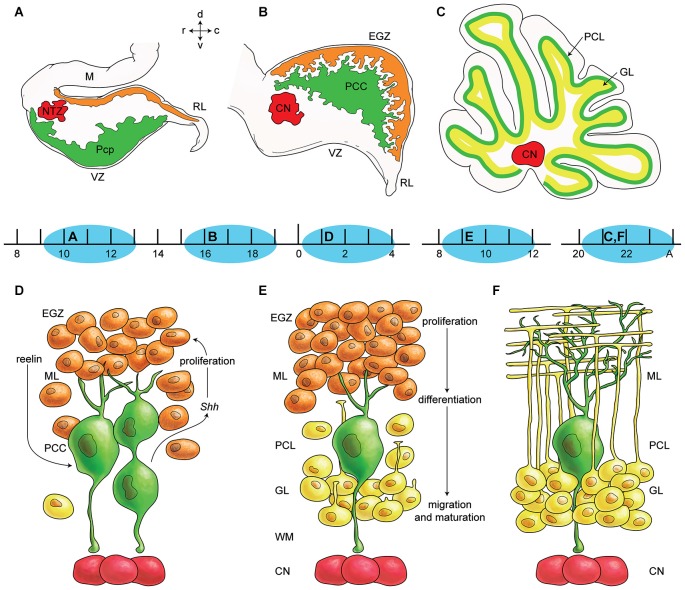
**Germinal zones in the developing cerebellum. (A–C)** Schematic illustration of the spatiotemporal parameters at sagittal sections of the early cerebellar development (embryonic (E) day 10–13 (E10–E13) **(A)**, E16–E17 **(B)**, and postnatal (P) day 20 (P20) to adult **(C)**. Neuroepithelium of 4th ventricle (ventricular zone (VZ)) is sources of all GABAergic neurons including Purkinje cells (green), Rhombic lip (RL) is sources of glutamatergic neurons including cerebellar nuclei neurons (red) and external germinal zone (orange; source of granule cells). **(D–F)** Schematic illustrations of the spatiotemporal parameters in corticogensis in which Purkinje cells cluster disperse in the monolayer and granular layer form. A cartoon of cerebellar cortex at around P4 **(D)**, at around P10 **(E)**, and in adult **(F)** is shown. Purkinje cells (green) express SHH that increases proliferative activity of external germinal zone (EGZ) cells (precursor of granule cells). Reelin express from precursor of granule cells and causes dispersal of Purkinje cells cluster **(D)** to monolayer **(E–F)**. Granule cells differentiate and migrate cross Purkinje cells layer to final destination i.e., granular layer and granule cells development is completed by maturation in this layer. Abbreviations: Pcc: Purkinje cell clusters, Purkinje cells precursor: pcp, mesencephalon: m, rhombic lip: RL, E: embryonic day, EGZ: external germinal layer (zone), gc: granule cells, m: mesencephalon, NTZ: nuclear transitory zone, A: Adult, pcl: Purkinje cell layer, RL: rhombic lip, ml: molecular layer.

The cerebellar primordium contains two germinal zones: the neuroepithelium of the fourth ventricle wall (ventricular zone—VZ) and the rhombic lip (Englund et al., 2006; Fink et al., 2006). For a long time, it was believed that all cerebellar neurons originate from the VZ, except granule cells that originate from RL (Altman and Bayer, 1985a,b,c; Wang and Zoghbi, 2001). However, recently it has been suggested that the rhombic lip also produces large projection cerebellar nuclei neurons and unipolar brush cells (Ben-Arie et al., 1997; Machold and Fishell, 2005). Secondary germinal zones include the external germinal zone (EGZ), rostral germinal zone (RGZ), and anterolateral border of the cerebellar plate (summarized in Figure 1). Transcription factor, protein, and receptor expression during cerebellar development and germinal zone specification has been reviewed recently (Garel et al., 2011; Hoshino, 2012). Two bHLH transcription factors, PTF1a and MATH1 might be essential in defining and specification of the VZ and the RL (Hevner et al., 2006; Hoshino, 2006a).

### Ventricular zone

All GABAergic cerebellar neurons are derived from the VZ of the cerebellar plate (Zhang and Goldman, 1996; Maricich and Herrup, 1999; Hoshino et al., 2005; Hibi and Shimizu, 2012). Purkinje cells are derived around E10–E13 in mouse (Miale and Sidman, 1961; Wang and Zoghbi, 2001; Hashimoto and Mikoshiba, 2003), while interneurons (stellate/basket and Golgi cells) are born postnatally (Miale and Sidman, 1961). Several transcription factors are important to instruct VZ cells to differentiate into GABAergic neurons (Hoshino et al., 2005; Hoshino, 2006a). *Notch1* mRNA is expressed in the cerebellar primordium as early as E9 and is restricted to neural progenitors in the VZ (Machold and Fishell, 2005). NOTCH regulates expression of a cascade of transcription factors belonging to the basic helix-loop-helix (bHLH) family (Kalyani et al., 1997; Robey, 1997; Artavanis-Tsakonas et al., 1999). Within the VZ, *Hes5* expression reflects NOTCH signaling activity in the cerebellar progenitor pool, while expression of DELTA1 likely indicates a subpopulation of neural precursors that are undergoing differentiation (Machold and Fishell, 2005). In addition, the bHLH transcription factor MASH1 appears to be expressed in precursors of GABAergic cerebellar neurons that arise from the VZ (Ma et al., 1997; Lutolf et al., 2002; Hoshino et al., 2005).

Recently, it was revealed that the VZ is characterized by *Ptf1a* expression. The mouse mutant cerebelless, which lacks the entire cerebellar cortex, has a mutation in the *Ptf1a* (Hoshino et al., 2005; Hoshino, 2006a; Butts et al., 2014). *Ptf1a* encodes a bHLH transcription factor, which was originally reported to be important in differentiation of endodermal cells into a pancreatic lineage. *Ptf1a* plays an essential role in generation of cerebellar GABAergic neurons; lineage-tracing analyses revealed that neuroepithelial cells in the cerebellar VZ produce neurons even in the absence of *Ptf1a* expression, although the subsequent neurons cannot differentiate into GABAergic neurons (Krapp et al., 1998; Kawauchi et al., 2005; Hoshino, 2006a; Millen et al., 2014). It seems that *Ptf1a* functions independent of the NOTCH signaling pathway (Beres et al., 2006).

### Rhombic lip; caudomedial germinal zone

The rhombic lip is a highly proliferative region of the neural fold located along the dorsal edge of the fourth ventricle in early development. It is present in all vertebrates (Wingate, 2001; Dun, 2012; Yeung et al., 2014). It can be subdivided into eight units according to the rhombomeres 1–8 (r1–8; r1 considered upper rhombi lip and r2–r8 considered lower rhombic lip), which are transiently recognizable during early developmental stages of the rhombencephalon (Altman and Bayer, 1997). Classically, the upper rhombic lip was thought to generate only cerebellar granule neurons (Altman and Bayer, 1997). Precursors are created from E12.5 to E17 and they migrate to establish the EGZ (Wingate, 2001; Machold and Fishell, 2005), which in turn gives rise to the granule cells during the first two postnatal weeks in mice (Miale and Sidman, 1961; Wang and Zoghbi, 2001). Recent studies indicate that other glutamatergic neuronal populations arise from RL (Machold and Fishell, 2005). These include projection neurons of the cerebellar nuclei, which originate at around E9–E12 (Miale and Sidman, 1961; Wang and Zoghbi, 2001; Fink et al., 2006; Millen and Gleeson, 2008; Yeung et al., 2014), and the unipolar brush cells, which are generated from E13.5 to the early neonatal period in mice (Hevner, 2006) and E15.5 to the neonatal period in rats (Mugnaini and Floris, 1994; Sekerková et al., 2004).

Rhombic lip derived *Math1* positive cells differentiate at the interface of VZ and roof plate. This depends both on DELTA-NOTCH signaling (from VZ) and direct contact with the Lmx1a- and Gdf7-positive non-neural cells of the roof plate (Butts et al., 2014). The *Math1* gene, which encodes a bHLH transcription factor, is expressed in glutamatergic cells of the rhombic lip as well as in proliferating granule cell precursors in the EGZ. Targeted disruption of this gene results in complete loss of the granule cell lineage and disruption of rhombic lip-derived cerebellar nuclei neurons (Wang and Zoghbi, 2001; Machold and Fishell, 2005; Fink et al., 2006). Recently it was shown that Wntless (*Wls*) is expressed in the interior face of the rhombic lip, complementary to and independent of *Math1*, which is localized to the exterior face of the rhombic lip (Yeung et al., 2014). *Barhl1* is a mouse homeobox gene that plays a role in cerebellum development. It is activated by the transcription factor MATH1, possibly in response to BMP. Expression of *Barhl1* mRNA was studied in human Carnegie stage 8, 12, 16, and 17 embryos (18 to 41 post-ovulatory days) and fetuses at 17, 22, and 24 weeks of gestation. *Barhl1* was restricted to the central nervous system, with strong expression in the rhombic lip at stage 17 and continued expression in the EGZ and granular layer to 24 weeks (Lopes et al., 2006).

In glutamatergic neurons,* Pax6* is downstream in the MATH1 pathway (Yeung et al., 2014), which includes sequential expression of *Tbr2*,* NeuroD*, and *Tbr1* (Stoykova and Gruss, 1994; Bulfone et al., 1995, 1999; Engelkamp et al., 1999; Lee et al., 2000). MATH1 regulates *Tbr1* expression (Wang et al., 2005) and is necessary for *Tbr2* and *Pax6* expression in the cerebellum (Hevner et al., 2006). Similarities between cerebrum and cerebellum suggest that these transcription factors may play conserved roles in a general program of glutamatergic neurogenesis. In the developing brain, Neurogenin2, a bHLH transcription factor, regulates glutamatergic differentiation of early-born neurons and expression of *Tbr1* and *Tbr2* mRNA (Schuurmans et al., 2004; Guillemot et al., 2006).

### External germinal zone

The external germinal zone (EGZ) is a temporary population of proliferating cerebellar cells located at the subpial surface of the developing cerebellum. The vast majority of cells from the EGZ produce granule cells during the postnatal period in rodents. In rats, the EGZ volume peaks at 15 days postnatal and disappears by 24 days (Heinsen, 1977). A subset of Golgi cells is also derived from EGZ (Chung et al., 2011). In the presence of both BMPs and Sonic hedgehog (SHH), a small proportion of the granule cell precursors differentiate into astroglial cells (Okano-Uchida et al., 2004). In rhesus monkeys, basket and stellate neurons differentiate adjacent to the EGZ (Rakic, 1972). Furthermore, the granule cell precursors in EGZ are attached to the basal lamina of external limiting membrane subpialy. The contact to the basal membrane apparently is an important factor of amplifying precursors cell in EGZ and may act corresponding to the cortical intermediate precursors in the subventricular (Butts et al., 2014).

The EGZ can be divided into an outer layer (proliferating zone) and an inner layer (post-mitotic granule cells). The post-mitotic granule cells develop axons that extend among the parallel fibers in the developing molecular layer while the somata migrate inward to the granular layer. The molecular signals involved in granule cell migration and differentiation have been reviewed recently (Chédotal, 2010; Furuichi et al., 2011).

SHH-signaling is an important driver of granule cell progenitor proliferation (Wechsler-Reya and Scott, 1999; Lewis et al., 2004; Haldipur et al., 2012). SHH secreted into the cerebrospinal fluid promotes precursor proliferation and regulates rhombic lip development and cell fate decision. Later in development, SHH acts on migrating cells in white matter (Butts et al., 2014). Purkinje cells secrete SHH starting at ~E18.5 in the mouse cerebellum (Corrales et al., 2004). *SHH* mRNA is detected in Purkinje cells and EGZ beginning at approximately 17 weeks gestation in human cerebellum (Aguilar et al., 2012). SHH immunoreactivity in Purkinje neurons is strong from 28 to 39 weeks and is absent by 4 months postnatal. Its receptors patched (PTC) and smoothened (SMO) and their effector GLI-2 exhibit similar temporal patterns of immunoreactivity in the EGZ (Corrales et al., 2004; Haldipur et al., 2011).

BMI1, which promotes cell proliferation, is expressed strongly in the cells of the EGZ at ~E16.5 during mouse cerebellar development. BMI1 is expressed in parallel with N-MYC and cyclin D2 in the EGZ of human cerebellum from 17 weeks gestation to 2 months postnatal (Leung et al., 2004).

Pituitary adenylate cyclase-activating polypeptide (PACAP) receptors appear to be important for cerebellar development in rodents. Basille et al. (2006) showed that the mRNA for receptors PAC1 and VPAC1 (but not VPAC2) and the PACAP binding capacity are abundant from 16 to 24 weeks gestation in the EGZ and granular layer. They disappear from EGZ after birth during the period of regression but increase in the granular layer continuing into adulthood. The data suggest that PAPAP might be neurotrophic in the EGZ, but other functions are implied in the mature granular layer.

*Zic* (zinc finger of the cerebellum) gene is expressed in the dorsal cranial neural tube and somites of E9.5 mouse. Granule cells and their precursors all express ZIC family members, even in adult stages in cerebellum (Houtmeyers et al., 2013). ZIC was detected by immunohistochemistry in the nuclei of EGZ and granular layer cells in cerebella from human fetuses at 21 and 37 weeks gestation and in a subpopulation of granular layer cells at 6 years postnatal (Yokota et al., 1996).

Granule cell precursor proliferation seems to be regulated by non-autonomous WNT and bone morphogenetic protein (BMP) pathway signals in the EGZ (Butts et al., 2014). It is suggested that cerebellar granule neurons require appropriate levels of WNT signaling to balance their proliferation and differentiation (Lorenz et al., 2011). Activation of the WNT/β-catenin signaling pathway results in severely inhibited proliferation and premature differentiation of cerebellar granule neuron precursors independent of BMP signaling (Butts et al., 2014). It seems WNT and BMP signaling pathways are antagonist to the SHH signaling pathway in the regulation of EGZ proliferation.

The pia mater also plays a role in granule cell proliferation and probably in inward migration along Bergmann glia. As granule cells mature, growing dendrites establish glomeruli (synaptic complexes) within the granule cell layer. This exuberant proliferative activity is accompanied by apoptosis, with dying cells scattered throughout the developing cerebellar cortex (Silbereis et al., 2010).

### Rostral germinal zone

Using quail-chick chimeras, Hallonet et al. demonstrated that the rostromedial end of the cerebellar primordium originates from caudal alar plate of the mesencephalon (Hallonet and Alvarado-Mallart, 1997). However, others reported that all cerebellar cells are born from *Otx2*-negative tissue (Millet et al., 1996) and thus the mesencephalic source remains a controversial issue. It has been shown during early development that *En1*/*En2*, *Wnt1*, *Fgf8* and *Acp2* are expressed in the caudal mesencephalon with extension to the rostral rhombencephalon (Millen et al., 1995; Hallonet and Alvarado-Mallart, 1997; Bailey et al., 2013). Point mutation in lysosomal acid phosphatase 2 (*Acp2*) leads to severe abnormalities in anterior cerebellum (Bailey et al., 2013, 2014). It has been suggested that mes/rhombencephalon junction in early development may be correspond to the location at lobule VI–VIII in the adult cerebellum (Hallonet and Alvarado-Mallart, 1997). Corticogenesis of the CZ (i.e., lobule VI/VII) is relatively delayed in mouse cerebellum (Marzban et al., 2008). In some species it can be indicated by a cortical area in paramedian sulcus of lobule VI–VII (e.g., bat (Kim et al., 2009); star-nosed mole (Marzban et al., 2014). Rostral and caudal to lobules VI–VII, the pattern of genes such as *En1*, *En2*, *Wnt-7* and *Gli* are differentially expressed (Hallonet and Alvarado-Mallart, 1997). In adult cerebellum, the striped pattern of gene expression is interrupted by the CZ (Marzban et al., 2003; Bailey et al., 2013). Mutation in some genes such as meander *Tail*—(Ross et al., 1990) and *Nax* mutant—(Bailey et al., 2014) causes defects in the anterior cerebellum, while mutation of other genes (e.g., *Lmx1a*) are associated with defects in the posterior cerebellum (Chizhikov et al., 2006b). In sum, current data indicate that the RGZ plays an essential role in development of anterior cerebellum and is governed by genes distinct from those in the caudomedial germinal zone.

### Anterolateral border of the cerebellar plate

The anterolateral border of the cerebellar primordium has no role as germinal zone, but does serve as a migratory route for precerebellar nuclei neurons from the rhombic lips. In addition, it provides a pathway for afferent and efferent axons prior to establishment of the cerebellar peduncles.

## Cell migration during cerebellum development

Migration of postmitotic neurons from their germinal location is a fundamental cellular event essential for building the nervous system (Wingate, 2001). In the developing cerebellum, neurons are born in multiple germinal zones and migrate to their destination using radial or tangential migratory pathways. Bergmann glia are specialized radial glia in the cerebellum (Yamada and Watanabe, 2002; Bellamy, 2006). They arise from the VZ, sending apical processes to the subpial surface and aligning their somata next to the Purkinje cell layer. Electron microscopic studies show Bergmann fibers in the EGZ by E15 in mice and E17 in rat (Del Cerro and Swarz, 1976), and by 9 weeks gestation in humans (Choi and Lapham, 1980). Immunohistochemical staining for tenascin shows radial glial fibers traversing the entire cerebellar primordium in E13 mice (Yuasa, 1996). Glial fibrillary acidic protein (GFAP) is expressed by human Bergmann glia as early as 12 weeks gestation (Bell et al., 1989). *Notch1*, *Notch2*, and *RBPj* genes play crucial roles in the development of Bergmann glia (Hiraoka et al., 2013).

In mouse cerebellum, Purkinje cell and other inhibitory cortical precursor cells exit the cell cycle in the VZ and migrate outward along the radial-glial-fiber system to establish a plate of immature neuronal cells in the middle of the anlage after E13 (Hatten and Heintz, 1995). The Purkinje cell plate is initially several cell layers thick and disperses into a monolayer after birth in mouse (Komuro and Yacubova, 2003; Xu et al., 2013) and by ~28 weeks gestation in human fetus (Zecevic and Rakic, 1976). Cortical inhibitory interneurons arise from the VZ and migrate outward to their final destinations in the molecular layer and the granule cell layer.

Proliferating cells derived from rhombic lip migrate rostrally to establish the EGZ. Continued proliferation of EGZ cells generates an extensive pool of precursors that spread subpially across the dorsum of the cerebellar anlage. The unipolar brush cells bypass subpial migratory pathway proximally and direct deep to the core of developing cerebellum, while large projection cerebellar nuclei neurons and granule cells migration continue rostrally in subpial stream pathway toward the distal (rostral) end of cerebellar primordium. In midway, the cerebellar nuclei neurons precursors change direction to reach the nuclei transitory zone (Figure 1).

Rhombic lip derivative precursors migrate tangentially independent to the glial guidance in subpial stream pathway (Wingate, 2001). The migrating cells exhibit a characteristic unipolar morphology where a single leading process appears to guide migration. Although molecular mechanism that induce granule cells switch on to their mode of migration in tangential and radial phases poorly understood, a number of candidate guidance molecules are expressed at the rhombic lip and EGZ such as ERBB4, UNC5H. Netrin has a general role in guiding migration of rhombic lip derivatives cell and in the formation of the EGZ (Wingate, 2001). Granule cell precursors express Unc5H receptors under the strict control of PAX6. Disruption of UNC5H3 signaling results in a rostral cerebellum malformation in which granule cells extend ectopically into the caudal mesencephalon. The roof plate-derived chemorepellent (such as BMP) may be involved in the migration probably by inducing *Math1* expression and directing the precursor processes away from the rhombic lip (Wingate, 2001; Butts et al., 2014).

Before their inward migration, within the outer layer of EGZ, granule cell precursors proliferate every 18–20 h and postmitotic cells remain in place for 24–48 h. During this postmitotic period the cells tangentially migrate to the inner layer of the EGZ and begin to extend their horizontal processes. At the interface of the EGZ and the molecular layer, the speed of migration is slowest and is accompanied by extension of processes into the molecular layer. In the molecular layer, the granule cells are elongated as they migrate radially along Bergmann glial processes, traversing the molecular layer in about 10–11 h to reach the Purkinje cell layer (Komuro and Yacubova, 2003). Here the cells separate from the Bergmann glial cells processes and their somata become spherical (Xu et al., 2013). Lamellipodia and filopodia at the distal portion of the leading process actively search for guidance cues. In the granule layer, granule cells migrate radially towards the bottom of the layer without any association with glial cells. Migration is completed when the tip of a leading process approaches the white-matter (Jiang et al., 2008).

Ca^2+^ signaling functions as a mediator for controlling granule cell migration (Komuro and Rakic, 1996); NMDA receptor activity significantly increases the rate of granule cell movement (Kumada et al., 2006). Molecules important for interaction with Bergmann glial processes include astrotactin, tenascin, fibronectin, neuregulin (NRG) and its receptor ERBB4, and the small G-actin-binding protein profilin1 (Rust et al., 2012).

The transcription factor CUX1 is an essential regulator in proliferating and migrating granule cells (Topka et al., 2014). Brain-derived neurotrophic factor (BDNF) positively regulates the proliferation, migration of granule cells. However, proBDNF negatively regulates granule cell migration mediated by P75 neurotropin receptor (Xu et al., 2011). In human fetal cerebellum, the EGZ cells express the low affinity nerve growth factor (NGF) receptor from 15 weeks gestation to 8 months postnatal and Purkinje cells are immunoreactive from 15–40 weeks gestation (Yachnis et al., 1993). The high affinity NGF receptors (TrkA, TrkB, TrkC) are expressed by a few EGZ and granular layer cells and by Purkinje neurons into adulthood (Quartu et al., 2003a,b).

Other molecules reported to be involved in granule cells migration include neurotrophin-3, stromal-cell-derived factor 1α (SDF-1α), ephrin-B2, tissue plasminogen activator, platelet-activating factor, cyclin-dependent kinase 5, and 9-O-acetyl GD3 (Kaslin and Brand, 2013; Butts et al., 2014). These activate distinct signaling pathways such as Ca^2+^ signaling, cyclic nucleotide signaling, and mitogen-activated protein kinase (MAPK) signaling.

**Figure 2:**
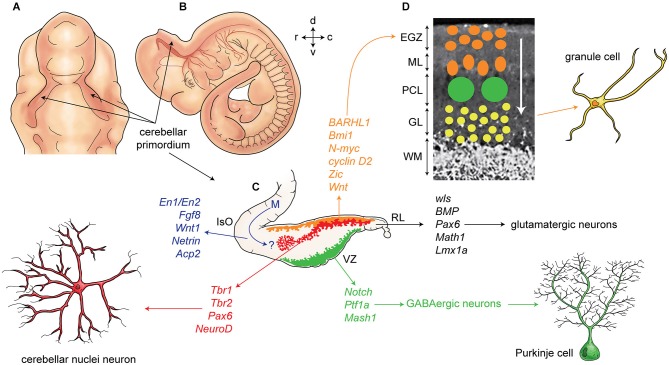
**Development of cerebellum and corticogenesis. (A–B)** Dorsal and lateral aspect of the mouse embryo at embryonic day E10-E11, showing outline of cerebellar primordium (indicated by arrows) and mesencephalon (midbrain) (m). **(C)** Schematic illustration of the developing cerebellum at about E11 indicates ventricular germinal zone (green) and genes are involved in neurogenesis of GABAergic neurons such as Purkinje cells. Rhombic lip is germinal zone that under control of genes such as *Wls, Bmp, math1, pax6,* and *Lmx1a* generate almost all glutamatergic neurons in cerebellum. Rhombic lip derived produce cerebellar nuclei neurons (red), and external germinal zones precursors (orange). An arrow from mesencephalon (m) indicates a germinal zone for group of cells derived from mesencephalon to the cerebellar primordium. **(D)** A section of cerebellum at around P4 indicate external germinal zone (EGZ) that after proliferation migrate through the Purkinje cells to the granular layer that is location of granule cells. Abbreviation; Iso: isthmic organizer, m: mesencephalon, EGZ: external germinal zone, ML: molecular layer, PCL: Purkinje cell layer, GL: granular layer, WM: white matter, 4thV: fourth ventricle, r: rostral, c: caudal, d: dorsal, v: ventral.

Finally, the extracellular glycoprotein, reelin, plays a critical role in cerebellar corticogenesis (summarized in Figure 2). In mice, reelin is expressed in the subpial stream of migrating rhombic lip derived cerebellar nuclei neurons at ~E13.5 and later in EGZ cells. Reelin promotes Purkinje cells precursor migration toward the pial surfaces (Miyata et al., 2010). Postnatally, reelin signaling triggers the dispersal of the Purkinje cells into the adult monolayer. Reelin binds with the Purkinje cell receptors very low-density lipoprotein receptor (VLDLR) and apolipoprotein E receptor 2 (APOER2). Reelin signaling is followed by phosphorylation of an intracellular cytosolic adaptor protein, DAB1 (Larouche et al., 2008; Miyata et al., 2010). However, *Reln* null mice have a small subset of Purkinje cells in monolayer suggesting that the reelin signaling pathway is not exclusively responsible for Purkinje cell migration.

## Apoptosis, autophagy and unfolded protein response

### Apoptosis and cerebellum development

**Figure 3:**
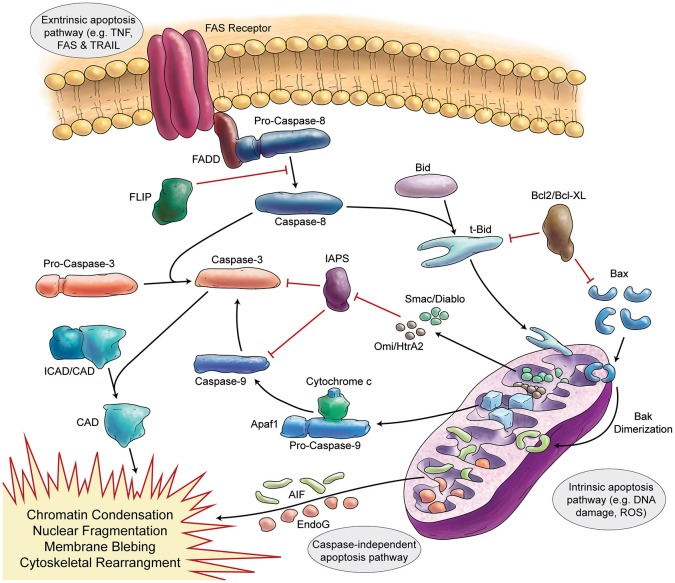
**Schematic Representation of Apoptosis**. In general apoptosis divides into extrinsic and intrinsic pathway. Death receptors (like FAS) are involved in extrinsic pathway, which later can activate caspase-8. Caspase-8 activates caspase-3 in two separate ways (direct activation or activation via caspase-9). Stress signals and DNA damage triggers intrinsic apoptosis pathway via mitochondria. Intrinsic apoptosis (mitochondrial apoptosis) is divided to caspase-dependent or caspase-independent pathways.

Programmed cell death (apoptosis) is a vital process present in different cell types, including neurons (summarized in Figure 3). The function of apoptosis in the developing nervous system seems to be the elimination of surplus neurons and establishment of correct synaptic connections (Catsicas et al., 1987; Oppenheim, 1991). Review articles make the extraordinary claim that approximately half of many types of neurons are eliminated (Johnson and Deckwerth, 1993; Raff et al., 1993). However, the primary data for cell death during mammalian cerebellar development have not been critically examined in detail.

The earliest descriptions of cell death in the developing cerebellum were based on morphologic features, typically nuclear pyknosis and fragmentation. In normal rats aged 1, 6, 12, and 21 days the mitotic index in the EGZ decreased from 2.7% to 0.7% and the pyknotic index ranged from 0.30 to 0.58%; the estimated total cell degeneration was 2.6% (Lewis, 1975). The ratio of dead cells to mitoses peaks at ~1% on day 10 in rats (Deo et al., 1979).The volume of the granular layer in rats peaks at ~25 days in rats, around the same time the EGZ disappears, and then diminishes by ~20%, reaching adult volume by ~50 days (Heinsen, 1977). Heinsen reported rare pyknotic cells but no phagocytosis and concluded that granular layer reduction was “mainly due to a decrease in the diameter of the granule cells and, to a lesser degree, perhaps to a distinct cell loss of these interneurons” (Heinsen, 1978). The pyknotic index in normal rat granular layer is <0.1% at 10, 14, and 21 days with dying cells most prevalent near the white matter at 10 days and near the Purkinje layer at 21 days (Rabié et al., 1977). Administration of ^3^H-thymidine to 9-day-old rats and subsequent analysis of mitotic activity and morphologic features of cell death led to the conclusion that the “decision to die” was made during the S phase of cell cycle and that a dead cell would be cleared in ~10 h (Deo et al., 1979). Administration of ^14^C-thymidine to 7, 12, and 16-day-old rats and subsequent analysis of tagged DNA 6 h or 5–9 days after administration showed no significant loss of DNA from the cerebellum at any of the ages. The authors concluded, “cell death, if it occurs at all, is probably less than 5% of total cells produced” (Griffin et al., 1978).

Caddy and Biscoe performed detailed neuron counts in mouse cerebellum and showed that the quantity of Purkinje cells increased from postnatal day 4 to 17 after which there was “no appreciable loss”. As was reported in rat, granule cells peaked at 17 days and then declined by ~20% by 26 days. Although they observed no morphologic signs of degeneration the authors did speculate on the role of programmed cell death (Caddy and Biscoe, 1979). It must be noted, however, that their data are based upon a single mouse at each time point (i.e., 6 total from 10 to 26 days) and therefore the result might not be reliable. In mouse cerebellum from 0 to 8 postnatal days the proportion of pyknotic (presumed dead or dying cells) was always <0.5%; most were located in the deep EGZ adjacent the molecular layer and it was estimated that they were eliminated in 4–7 h (Smeyne and Goldowitz, 1989).

More recently, “specific” markers of apoptotic cells have typically been used to study cell death. Wood and coworkers claimed that detection of T7 DNA polymerase was a valid marker of apoptosis-associated DNA fragmentation. In mouse cerebellum examined from postnatal day 5 to 30 they reported labeling in “scattered” EGZ cells at day 5, dense labeling at day 7, reduced labeling at day 9, and negligible labeling at day 21 (Wood et al., 1993). However, inspection of their images shows considerable background making unambiguous identification difficult. Furthermore, the detection method has not been used widely and therefore must be considered of limited value.

Using the TUNEL method, many investigators have confirmed the presence of dying cells in the immature mouse cerebellum, but there is a wide range in the reported quantities and considerable variability in the reporting methods. Earlier studies tended to report relative values per unit area rather than as proportions of total cells. In C57BL mice at 9 days only few cells (<10 per 2 mm length of EGZ and <5 per 2 mm of granular layer) were TUNEL labeled (Wullner et al., 1995). A more recent study reported ~55000 TUNEL positive cells per mm^3^ of EGZ in 9 day mouse cerebellum (Kubera et al., 2012). Where TUNEL positive cells in the mouse cerebellum have been counted, the proportion is typically low; e.g., <0.5% at 10 days in the EGZ (McNamee et al., 2002). In 3-day-old mice, only rare Purkinje neurons are immunoreactive for activated (cleaved) caspase 3 (and presumably undergoing apoptosis); these tend to be surrounded by microglia (Marín-Teva et al., 2004). Only rare cells in the day 7 mouse EGZ are positive for activated caspase 3 (Cabrera et al., 2014). In a clearly quantified study of activated caspase 3 immunoreactivity in mouse cerebellum from birth to 70 days, Cheng et al. reported peak labeling of 5.9% in the EGZ at 8 days, 5.9% in the Purkinje layer at 3 days, and 2.4% in the granular layer at 9 days (Cheng et al., 2011). In the granular layer of 7 days mice, activated caspase 3 counts are approximately double the TUNEL counts (Taranukhin et al., 2010). One group has reported that cleaved caspase 3 might have non-apoptotic functions in developing rat Bergmann glia; therefore without colocalization studies, reported values of caspase 3 might overestimate the number of apoptotic cells (Oomman et al., 2005; Finckbone et al., 2009). The same group reported that cleaved caspase 3 immunoreactivity in the EGZ of day 8–17 rat cerebellum has no association with TUNEL (Oomman et al., 2004), however the nearly ubiquitous labeling seems potentially artifactual despite the authors’ extensive controls.

Tanaka and Marunouchi studied rat cerebellum from 3 to 15 postnatal days and observed the greatest number of TUNEL positive EGZ cells at 9 days. Double label studies led them to conclude that most dying cells were in the proliferative phase rather than post mitotic (Tanaka and Marunouchi, 1998). In 3-day rat cerebellum, TUNEL positive cells were most abundant in the granular layer (~4 cells/mm^2^ tissue area) and were much less frequent than mitotic cells that had incorporated bromodeoxyuridine (~1200 cells/mm^2^ tissue area) (Vidovic et al., 2008). Krueger et al. reported in rats that at day 7 almost half the cells in white matter are TUNEL positive and most appear to be astrocytes. Despite their claim to do a similar study on granule cells, those data do not appear to have been published (Krueger et al., 1995). Quantitation of TUNEL in rat cerebella showed <1% of cells at 14 and 21 days and no labeling at 28 days, although the authors did not specify the anatomical localization of the positive cells (Wang et al., 2012b). Another group reported ~1% of granular layer cells to be TUNEL positive in 16 day rats (Sinha et al., 2009).

Lossi and coworkers (Lossi et al., 2002a,b; Lossi and Merighi, 2003) studied cell death in the rabbit cerebellum at day 5 postnatal, when EGZ proliferation is maximal, using a variety of markers. TUNEL labeling was not counted, but one of their images (Figure 1E; Lossi et al., 2002b) shows an estimated 5% cells positive, especially in the outer proliferative region of the EGZ. Apoptotic cells are less abundant in the granular layer, peaking at day 10. Most cells appear to die within 12–24 h after proliferation and are subsequently engulfed by microglia. Cleaved caspase 3, 7, and 9 immunostaining is almost exclusively restricted to the granular layer while the substrate of activated caspase 3, poly-ADP-ribose polymerase-1 (PARP-1), is mainly identified in the EGZ (Lossi, 2004).

In summary, the data from human (considered in an earlier section) and animals (especially rodents) indicate that cell death occurs in the developing cerebellum to a limited extent, particularly in the EGZ among recently postmitotic cells and to a lesser extent in the granular layer. The reported extent to which this occurs varies widely, but overall it seems reasonable to estimate that a maximum 5% of granule cells and a smaller proportion of Purkinje cells are lost, although it must be noted that studies in the very early developmental stages are lacking. Even the so-called “specific” methods of TUNEL and activated caspase 3 detection have limitations and must be interpreted cautiously. Nevertheless, the fact is that some of the cells do die and therefore the molecular mechanisms are worth considering.

Many *in vitro* models have been used. Cerebellar granule cells undergo apoptosis when they are deprived of extracellular potassium (D’Mello et al., 1993). Transforming growth factor (TGF-β) β1, -β2, and -β3 accelerate apoptosis of these neurons when maintained in low physiological potassium medium as assessed the quantitative DNA fragmentation, viability, and nuclear morphology (de Luca et al., 1996). A P53-independent apoptotic pathway has been proposed for loss of cerebellar granule cells during development. Neuronal precursors still undergo apoptosis in the cerebellum of transgenic mice that lack functional p53 (Wood and Youle, 1995).

### Autophagy and cerebellum development

**Figure 4:**
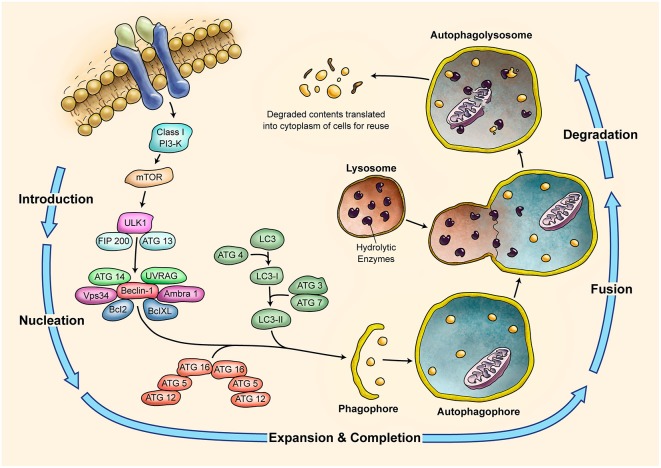
**Schematic Representation of Autophagy Pathway**. Autophagy is recognized as a major tool to degrade damaged organelles and misfolded proteins via lysosomal pathway. Autophagy is an active flux which includes five different steps; introduction or initiation, phagosome nucleation, phagosome expansion and completion, phagosome and lysosme fusion (autophagolysosome formation), and finally degradation. It is a tightly regulated mechanism and several *ATG* molecules are included in its regulation.

Autophagy is a self-degradative lysosomal process used for degrading and recycling various cellular constituents (summarized in Figure 4; Klionsky, 2005; Massey et al., 2006; Yang and Klionsky, 2010). During development cells undergo phases of both quiescence and enhanced metabolism; therefore, they require the ability to change their protein content to rapidly adapt to adverse conditions. Autophagy could help renovate cells or modify their morphology (Cecconi and Levine, 2008). The complex ontogenesis and development of nervous system is especially sensitive to dysregulation of autophagy. An example is shown by the *ULK1*^−/−^ (*UNC-51*-like kinase 1) mice phenotype. The deficiency in *ULK1*, an autophagic protein being involved in autophagosome initiation, causes abnormal growth cone and axon formation in the cerebellar granule neurons (Tomoda et al., 1999). The regulation of autophagy by *ULK1* in the neurodevelopment remains to be understood (Di Bartolomeo et al., 2010). *Atg7* (autophagy related 7) deficiency in mice is associated with severe neuronal loss from the cerebellar cortices (Komatsu et al., 2006). Genetic ablation of *Atg7* causes dystrophy of Purkinje cell axon terminals in the deep cerebellar nuclei (DCN). Selective ablation of *Atg5* or *Atg7* genes in neurons leads to behavioral deficits associated with neuronal loss in the cerebellar cortices (Komatsu et al., 2006, 2007a,b). Axon terminal degeneration is observed when *Atg7* is inactivated in the subpopulation of Purkinje cells (Komatsu et al., 2007b). Together with the *ULK1*-inactivation phenotype, the axon terminals seem to be very susceptible to autophagy impairment. The *Apaf1* is a gene involved in the formation of apoptosome. When it is down regulated *in vivo*, cells of the cerebellar cortex undergo the autophagy (Moreno et al., 2006). These experiments suggest that autophagy is constitutively active and is a mandatory process for the survival and development of cerebellar cells (Rami, 2009).

### Unfolded protein response (UPR) and cerebellar development

**Figure 5:**
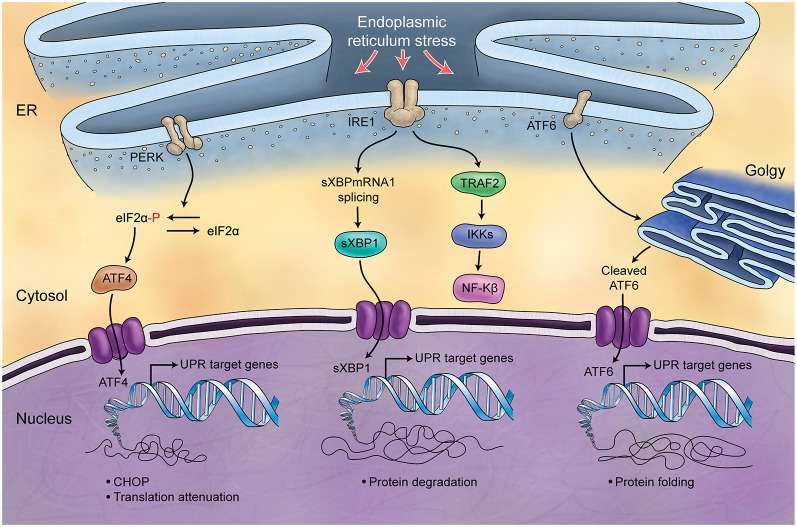
**Schematic Representation of Unfolded Protein Response**. Endoplasmic reticulum (ER) is involved in the processing of proteins and is responsible for regulation of protein folding. ER chaperones (PERK, IRE1, ATF6) are deactivated in normal conditions while in stress conditions and increase of misfolded proteins in ER, they will be activated (UPR) and differently control protein biosynthesis, cell survival, protein translation and cell cycle.

The endoplasmic reticulum (ER) is responsible for the synthesis and folding of proteins entering the secretary pathway (summarized in Figure 5). Many post-translational modifications that ensure protein function occur in this organelle (Denzel et al., 2002; Luo et al., 2006). A variety of physiological perturbations can interfere with processes of protein folding in the ER lumen, leading to the unfolded or misfolded protein accumulation, which is called “ER stress.” This stress triggers and activates an adaptive reaction, termed UPR, through which protein folding capacity increases and unfolded protein load decreases (Ni and Lee, 2007). These pathways of protein quality control are crucial for Purkinje cell survival (Zhao et al., 2005; Hara et al., 2006; Lee et al., 2006), and are related to the Marinesco-Sjögren Syndrome in humans (Anttonen et al., 2005; Senderek et al., 2005). A few studies directly support the role of UPR in the developing process of cerebellum. ORP150 is an ER protein that is upregulated in Purkinje cells during cerebellar development. Overexpression of ORP150 protein in Purkinje cells reduces the vulnerability of these cells to hypoxic and excitotoxic stress and increases their survival during cerebellar development (Kitao et al., 2004). BAP (SIL1) gene is another regulator of UPR, acting as a co-chaperone. Defects in BAP may result in Purkinje cell degeneration and ataxia (Anttonen et al., 2005; Senderek et al., 2005; Zhao et al., 2005, 2010). It is noteworthy that the cerebellum is particularly sensitive to BAP function loss and this could be explained by the observation that in the unaffected cerebellar lobules other co-chaperones can compensate for BAP function. Genetic manipulation of the GRP78 chaperone indicates that it too is very important for cerebellum development. Knock-in mice with a mutant secreted form of GRP78 show disordered layer formation in the cerebral cortex and cerebellum (Impagnatiello et al., 1998). It can be concluded that UPR potentially affects the development of cerebellum; however, there is still much room for elucidation of its role(s).

## Epigenetic mechanisms and cerebellar gene expression

### Epigenetic mechanisms

Epigenetics refer to cellular mechanisms that control gene expression without directly altering the underlying genomic sequence (Liyanage et al., 2012). Epigenetic mechanisms include DNA methylation, the activity of non-coding small RNA molecules such as microRNAs (miRNAs) and long non-coding RNAs, in addition to histone post-translational modifications (Delcuve et al., 2009). Epigenetic mechanisms are continuous, dynamic and in many occasions reversible processes that are involved in development (Rastegar et al., 2004; Nolte et al., 2006; Barber and Rastegar, 2010), regulation of developmentally important genes (Lahuna et al., 2000; Rastegar et al., 2000a,b; Barber et al., 2013), stem cell differentiation (Huang et al., 2005; Kobrossy et al., 2006; Olynik and Rastegar, 2012), and human diseases (Sandhu et al., 2012; Zachariah and Rastegar, 2012). Several neurological disorders are caused by mutations in the genes encoding for epigenetic factors. Perhaps the most studied neuronal disorder that is linked to epigenetic factors is Rett Syndrome, a severe neurodevelopmental disorder caused by *MECP2* (Methyl CpG Binding Protein 2) gene mutations. *MECP2* gene encodes for a key epigenetic regulator in brain that binds to methylated DNA (Meehan et al., 1992; Liyanage and Rastegar, 2014) and its own expression is also controlled by DNA methylation (Liyanage et al., 2013; Olson et al., 2014). While MeCP2 has a wide expression pattern, its highest expression is detected in the brain and most MeCP2-associated diseases are neurological disorders (Ezeonwuka and Rastegar, 2014). Other examples include mutations in the *ATRX* gene, which encodes for a chromatin-remodeling factor and these mutations are associated with X-linked mental retardation syndromes (Gibbons and Higgs, 2000). Several studies have indicated that in such disorders, exogenous expression of the causative gene product or its downstream targets may lead to phenotypic rescue both *in vitro* and *in vivo* (Giacometti et al., 2007; Rastegar et al., 2009; Cobb et al., 2010).

### Role of imprinting in cerebellar development

Genomic imprinting is an epigenetically regulated cellular mechanism by which expression of specific genes are regulated in a parent-of-origin manner. Two such genes, *H19* and insulin-like growth factor 2 (*Igf2*), are imprinted in a reciprocal manner by methylation of specific alleles within the *Igf2/H19* imprinting control region (ICR). The *Igf2/H19* ICR contains four CTCF binding sites, which are methylation sensitive and capable of regulating the interaction between the promoter of *Igf2* and enhancers located downstream of the *H19* gene (Pidsley et al., 2012b). In both humans and mouse, DNA methylation of *Igf2/H19* ICR correlates with the weight of the cerebellum (Pidsley et al., 2012a,b). Moreover, in humans, alleles inherited maternally are associated with an increase in the weight of the cerebellum as compared to paternally inherited alleles (Pidsley et al., 2012a).

### DNA methylation and cerebellum

DNA methylation is an epigenetic modification that involves a covalent addition of a methyl group to cytosine residues (5 methylcytosine; 5mC), mostly in the context of CpG dinucleotides (Liyanage et al., 2014). DNA methylation can regulate gene expression directly, by inhibiting the binding of specific transcription factors, or indirectly by the recruitment of factors that bind to methylated DNA, such as MeCP2. Another epigenetic modification that has recently attracted much attention is DNA hydroxymethylation (5hmC) (Liyanage et al., 2014).

DNA methylation profiling across various brain regions have indicated that cerebellar genes are hypomethylated in comparison to prefrontal, occipital, and temporal regions of the cortex. Consequently, genes within the cerebellum show higher expression levels as compared to the cerebral cortex (Xin et al., 2010). Within the cerebellar granule cells, 5hmC levels have been shown to increase between P7 to adult in mice. As compared to the hippocampus, cerebellum also showed a significant increase in 5hmC levels from 6 weeks to one year, suggesting brain region specific age-related alterations in 5hmC levels (Szulwach et al., 2011). In both mouse and human cerebellum, 5hmC-enriched loci were mostly intragenic, with greater enrichment being evident on exons than introns. Enrichment of 5hmC levels were also observed in short interspersed class elements. However, in contrast to mouse cerebellum, there was a lack of significant enrichment on long terminal repeats (Szulwach et al., 2011).

Changes in cerebellar hydroxymethylation have been associated with specific disorders. One of the first neuronal disorders to be associated with cerebellar hydroxymethylation was Rett Syndrome, caused by mutations in the X-linked *MECP2* (Amir et al., 1999; Liyanage and Rastegar, 2014). Alternative splicing of *MEPC2/Mecp2* leads to the formation of two isoforms, MeCP2E1 and MeCP2E2 (Kriaucionis and Bird, 2004; Mnatzakanian et al., 2004; Zachariah et al., 2012; Olson et al., 2014; Yasui et al., 2014). Comparative studies on the expression of the two isoforms have revealed differential expression patterns in various brain regions, with MeCP2E2 showing selective enrichment in the cerebellum and olfactory bulb (Zachariah et al., 2012; Olson et al., 2014). Szulwach et al. demonstrated that cerebellar 5hmC levels exhibit negative correlation with *Mecp2* dosage in mouse models of Rett Syndrome and *MECP2* duplication (Szulwach et al., 2011).

Altered cerebellar 5hmC levels have been associated with fragile X syndrome, a monogenic autism spectrum disorder. Fragile X syndrome is caused by mutations leading to the expansion of a CGG repeat within the 5′ untranslated region of fragile X mental retardation gene 1 (*FMR1*) (Darnell and Klann, 2013). The resulting expansion leads to DNA methylation-mediated transcriptional silencing of Fragile X mental retardation protein (FMRP). A comparison of 5hmC levels in FMRP targets within cerebellum revealed an enrichment of 5hmC in FMRP targets. FMRP targets also showed strong association with both stable and dynamic hydroxymethylated DNA, suggesting a critical role of 5hmC in FMRP function (Wang et al., 2012a).

Another disorder associated with the *FMR1* gene is Fragile X-associated tremor/ataxia syndrome (FXTAS), a neurodegenerative disorder associated with expansion of multiple CGG repeats (premutations) located within the gene (Hagerman et al., 2001). FXTAS leads to Bergmann gliosis, Purkinje cell death and cerebellar degeneration (Greco et al., 2006). Yao et al. observed a global reduction of 5hmC in the cerebellum of a mouse model of FXTAS. Analysis of the alteration in 5hmC distribution within the same mouse model revealed that the reduction of 5hmC was within the gene body. However, increased 5hmC levels were observed in several repetitive classes, including simple repeats, long terminal repeats and short interspersed nuclear elements (Yao et al., 2014). These studies indicate the significance of DNA methylation, 5hmC levels, and epigenetic modifications for cerebellar development and function that warrant further investigation for the role of 5mC and 5hmC in other cerebellar disorders.

### Role of miRNAs in cerebellar development

miRNAs are small non-coding regulatory RNAs that are found ubiquitously in animal cells. miRNAs are usually encoded in the introns of coding regions or within intergenic regions. One single miRNA may have numerous target genes, and therefore can be very potent gene regulatory mechanisms. The functions of miRNAs have been mostly correlated to the negative regulation of gene expression (Carthew and Sontheimer, 2009; Eulalio et al., 2012). miRNAs are known to regulate several transcription factors that are critical for normal brain developmental and function and altered expression of miRNAs have been linked to several neuronal disorders (Follert et al., 2014; Sun and Shi, 2014). Several groups have reported enrichment of specific miRNAs within the cerebellum. For instance, Olsen et al. reported *miR-206* and *miR-497* being expressed at significantly higher levels in the adult cerebellum compared to other brain regions in rats. The same group reported that *miR-221* family members (*miR-221* and *miR-222*) are selectively reduced compared to other brain regions such as amygdala and hippocampus (Olsen et al., 2009). In mice, on the other hand, Bak et al. reported that *miR-195*, *miR-497*, and *miR-30b* are enriched in the mouse cerebellum (Bak et al., 2008) whereas Hohjoh and Fukushima reported cerebellar enrichment of *miR-16*, -*miR-34a*, in addition to *miR-195* (Hohjoh and Fukushima, 2007).

The critical role of *miRNAs* in cerebellar development was elegantly demonstrated by Schaefer et al., who reported that cell-specific ablation of *Dicer*, an endonuclease necessary for miRNA generation, results in cerebellar Purkinje cell death as well as degeneration of the cerebellum (Schaefer et al., 2007). Phenotypically, ablation of *Dicer* specifically in Purkinje cells leads to an ataxic gait, indicative of cerebellar dysfunction. In 2011, Tao et al. demonstrated that deletion of *Dicer* in astroglia leads to widespread granule cell apoptosis and degeneration of Purkinje cell dendrites at a late postnatal stage (P55–P65) (Tao et al., 2011). The same study reported that deletion of *Dicer* altered the cerebellar astrocytic transcriptome at a pre-symptomatic stage, causing selective downregulation of genes associated with mature astrocyte functions, with the inverse effect on genes related to immature/reactive astrocyte genes. Another group also reported that deletion of *Dicer1* disrupts the phenotype of Bergmann glia within the cerebellum and decreases the expression of its markers (Kuang et al., 2012). These studies suggest that optimal miRNA generation by both astrocytes and neurons are necessary for proper cerebellar function and development.

### Cerebellum involvement in autism and the associated epigenetic control

Abnormalities in the cerebellum have been reported in more than 95% of post mortem examinations of autistic individuals (Delong, 2005). A reduction in the number of Purkinje neurons is the most widely reported neuropathology, along with cerebellar hypoplasia, (Courchesne et al., 1988; Courchesne, 1997; Palmen et al., 2004; DiCicco-Bloom et al., 2006). Stereological analysis of Purkinje cells in the cerebellum of 14 autistic individuals age 4 to 60 years showed a 25% reduction in number and a 24% reduction in density compared to controls (Wegiel et al., 2014). *In vivo* studies by MRI in autistic children have also shown a reduction in the cerebellar vermal volume (Webb et al., 2009). Decreased cerebellar volume has been reported in Asperger’s syndrome and Rett syndrome, two disorders sharing many overlapping phenotypes with classical autism (Oldfors et al., 1990; Murakami et al., 1992; Hallahan et al., 2009).

At the molecular level, several genes with known roles in cerebellar development are associated with autism. Examples of such genes include engrailed 2 (*EN2*) and mesenchymal-epithelial transition (*MET*) receptor tyrosine kinase (Fatemi et al., 2012; Rogers et al., 2013). In animal models, altered expression of *En2* and *Met* has been associated with reduction in the cerebellar volume and cerebellar hypoplasia (Ieraci et al., 2002; Kuemerle et al., 2007; Provenzano et al., 2014). *EN2* expression is known to be epigenetically regulated by multiple mechanisms including DNA hydroxymethylation (James et al., 2014) and miRNAs (Guibinga et al., 2012). A recent study on cerebellum samples from autistic patients indicated that increased 5hmC levels on *EN2* promoter correlated with its increased expression. This study also reported a global increase in 5mC and 5hmC levels in the cerebellum of autistic patients (James et al., 2014). Furthermore MeCP2, a well-characterized epigenetic regulator, controls the allele-specific expression of *MET* (Plummer et al., 2013). These studies highlight the complex mechanisms by which epigenetic regulatory networks contribute to autistic phenotypes.

### Mouse models of cerebellum maldevelopment

Given the complexity of the human brain and the many genes that function during neural development, it is not surprising that we currently have a limited understanding of the mechanisms of brain diseases. Spontaneous mutations along with engineered gene knockouts and transgenic mice provided important models to study cerebellum. During the last several decades, mutant mice have significantly improved our knowledge of cerebellar development, cytoarchitecture, function, and disease. The selenoproteins are important in postmitotic neurons of the developing cerebellum, and mutations in these genes cause cerebellar hypoplasia as occurring in neurodevelopmental syndrome called progressive cerebello-cortical atrophy (PCCA; Wirth et al., 2014). SDF-1/CXCR4 signaling plays an important role in neuronal cell migration and brain development. Mutation in *Cxcr4* gene causes neurodevelopmental disorder of granule cells and Purkinje cells that leads to the motor defects in null mice (Huang et al., 2014). Animal models for autism spectrum disorder and schizophrenia that selectively target the cerebellum has been recently reviewed (Shevelkin et al., 2014).

Mutant mice are useful models for rescue experiments that might lead to significant future applications of gene therapy (Marmolino and Manto, 2010; Sajan et al., 2010). Spontaneous mutant and recent transgenic mouse models whose main characteristics are cerebellar defects are summarized in Table 2.

**Table 2:** **Mutant and transgenic mice with cerebellar abnormalities and relevant human disease**.

Mouse model/gene	Function	Developmental defect	Relevant	References human disease
*Staggerer*/RORα	Retinoid-related orphan receptor α	Small cerebellum, Purkinje and granule cells degeneration	NA	Boukhtouche et al. (2006), Gold et al. (2007), Sajan et al. (2010)
*Lurcher/Grid2*	Delta 2 ionotropic glutamate receptor	Degeneration of Purkinje, granule cells	NA	Zuo et al. (1997), Armstrong et al. (2011)
*Leaner/Cacna1a*	Voltage-dependent calcium channels	Purkinje and granule cell death	NA	Herrup and Wilczynski (1982)
*Weaver/Girk2*	Potassium inwardly rectifying channel	Purkinje and granule cell death	NA	Patil et al. (1995)
*Reeler/Reln*	Main factor of neuronal migration	Small cerebellum with no foliation and ectopic clusters of Purkinje cells	Lissencephaly	Miyata et al. (2010)
*Scrambler/Dab1*	Main regulator of reelin signaling pathway	Small cerebellum with no foliation and ectopic clusters of Purkinje cells	NA	Goldowitz et al. (1997)
*Dreher/Lmx1a*	LIM homeobox transcription factor 1, alpha	Posterior cerebellar defect/ mainly vermis hypoplasia	Dandy walker syndrome (Possible)	Chizhikov et al. (2006b)
*Nax/Acp2*	Lysosomal acid phosphatase 2	Neurocutaneous disorder/small cerebellum, severe anterior cerebellar disorder with an absent or hypoplastic vermis	GLHS (Possible)	Mannan et al. (2004), Bailey et al. (2014)
*Cerebelless/ptf1a*	Pancreas transcription factor 1, alpha	Lacks the cerebellar cortex	Cerebellar agenesis	Hoshino (2006a)
*Cux1*	Transcription factor	Granule cell precursors proliferation	Medulloblastoma	Topka et al. (2014)
*SEPSECS*	Selenoproteins; selenium transferase	Uncoordinated movements, cerebellar hypoplasia, Purkinje cell death and decreased granule cell proliferation.	Progressive cerebello-cortical atrophy (PCCA)	Wirth et al. (2014)
*CXCR4*	C-X-C chemokine receptor type 4	Purkinje cell dendritogenesis and axonal projection	NA	Huang et al. (2014)
*CHD7*	Chromodomain-helicase-DNA-binding protein 7	Alteration of Otx2, Gbx2 and fgf8 and cerebellar vermis development	CHARGE syndrome	Yu et al. (2013), Basson (2014)
*CACNA1A*	Voltage-gated calcium channel subunit α1A	Ataxia and cerebellar atrophy in transgenic mice	Spinocerebellar ataxia type 6 (SCA6)	Du et al. (2013)
*CARP VIII/*	Carbonic anhydrase-related protein VIII	Ataxia	Mental retardation and ataxia	Aspatwar et al. (2013)
*CHMP1A*	Chromatin modifying protein 1A	Pontocerebellar hypoplasia	Pontocerebellar hypoplasia	Mochida et al. (2012)
*PDSS2*	Decaprenyl-diphosphate synthase subunit 2; ubiquinone biosynthesis	Cerebellum hypoplasia by impairing cell migration and eliciting ectopic apoptosis	Ubiquinone deficiency in humans	Lu et al. (2012)
*Fgf17*	Fibroblast growth factors	Cerebellar vermis abnormalities	Dandy-Walker malformation	Zanni et al. (2011)
*ZIC1; ZIC4*	Transcription factors; zinc fingers in cerebellum	Cerebellar size and foliation	Dandy-Walker malformation	Blank et al. (2011)

Medulloblastoma, a tumor typically involving the cerebellum, is the most common high-grade brain tumor in childhood. Four morphologic subtypes correspond to different genetic signatures. A detailed review is beyond the scope of this manuscript, but it is worthwhile mentioning the postulated cell populations of origin for these tumors: Wnt subtype might arise from the lower rhombic lip progenitors, SHH subtype from the precursor cells of the EGZ, group 3 subtype from prominin1 expressing neural stem cells or EGZ, and group 4 subtype from an unknown population of stem or progenitor cells. Readers are referred to recent comprehensive reviews for more details (Manoranjan et al., 2013; Gajjar and Robinson, 2014; Wang and Wechsler-Reya, 2014).

## Summary

Anatomically, the cerebellum development begins at around E8 to E9 and cerebellar germinal zones are established during E9 to E13–14 in mice and the late embryonic period in humans The germinal zone can be categorized in four groups: internal germinal zone (VZ), EGZ, caudomedial germinal zone (rhombic lip) and RGZ (mesencephalon). VZ is the source of Purkinje cells as well as all GABAergic interneurons in addition to a subset of non-neuronal cells. The EGZ is the source of all granular cells and some interneurons (such as Golgi) and non-neuronal cells. Granular cell are generated in the postnatal period of both humans and rodents. Many genes and signaling pathways are involved in the development, cell fate commitment, and migration of these cells in the cerebellum. Cell death occurs among a limited number of EGZ and granular cells. Recent studies have shown a critical role for epigenetic factors in cerebellum development. This includes DNA methylation and specific miRNAs in controlling various neural processes. The necessity of miRNAs for the survival of cerebellar neurons underscores its importance in cerebellar development. Furthermore, the various forms of DNA methylation are being recognized for their multifaceted role in regulating the precise expression levels of transcription factors essential for normal cerebellar function. The significance of these regulatory mechanisms has been further elucidated by studies that have linked several neurological disorders to alterations in epigenetic control of cerebellar gene expression.

## Conflict of interest statement

The authors declare that the research was conducted in the absence of any commercial or financial relationships that could be construed as a potential conflict of interest.

## Abbreviations

5hmC: 5 hydroxymethylcytosine
5mC: 5 methylcytosine
CZa: Anterior central zone
AZ: Anterior zone
ATG: Autophagy-related genes
APOER2: Apolipoprotein E receptor 2
bHLH: Basic helix-loop-helix
BMP: Bone morphogenetic protein
BDNF: Brain-derived neurotrophic factor
CZ: Central zone
CMA: Chaperone-mediated autophagy
DCN: Deep cerebellar nuclei
DON: Dorsal octavolateral nucleus
ER: Endoplasmic reticulum
EGZ: External germinal zone
FGR: Fetal growth restriction
FXTAS: Fragile X-associated tremor/ataxia syndrome
FMRP: Fragile X mental retardation protein
fog: Forebrain overgrowth
GFAP: Glial fibrillary acidic protein
ICR: Imprinting control region
*Igf2*: Insulin-like growth factor 2
*Irxs*: Iroquas
*MECP2*: Methyl CpG Binding 2 human gene
*Mecp2*: Mouse gene
MeCP2: protein
*MET*: Mesenchymal-Epithelial Transition
MON: Medial octavolateral nucleus
miRNA: micro RNA
NGF: Nerve growth factor
NRG: Neuregulin
NZ: Nodular zone
PTC: Patched
PACAP: Pituitary adenylate cyclase-activating polypeptide
CZp: Posterior central zone
PZ: Posterior zone
PCCA: Progressive cerebello-cortical atrophy
RL: Rhombic lip
RGZ: Rostral germinal zone
SMO: Smoothened
TET: Ten-eleven translocation
SHH: Sonic Hedgehog
SDF-1α: Stromal-cell-derived factor 1α
TUNEL: terminal deoxynucleotidyl transferase-mediated dUTP nick end labeling
TGF: Transforming growth factor
VZ: Ventricular zone
VLDLR: Very low-density lipoprotein receptor
Wls: Wntless
Zic: zinc finger of the cerebellum.

## References

[B1] Abrahám H. Tornóczky T. Kosztolányi G. Seress L. (2001). Cell formation in the cortical layers of the developing human cerebellum. Int. J. Dev. Neurosci. 19, 53–62. 10.1016/s0736-5748(00)00065-4 11226755

[B2] Aguilar A. Meunier A. Strehl L. Martinovic J. Bonniere M. Attie-Bitach T.. (2012). Analysis of human samples reveals impaired SHH-dependent cerebellar development in Joubert syndrome/Meckel syndrome. Proc. Natl. Acad. Sci. U S A 109, 16951–16956. 10.1073/pnas.1201408109 23027964 PMC3479472

[B3] Alder J. Lee K. J. Jessell T. M. Hatten M. E. (1999). Generation of cerebellar granule neurons in vivo by transplantation of BMP-treated neural progenitor cells. Nat. Neurosci. 2, 535–540. 10.1038/9189 10448218

[B4] Altman J. Bayer S. A. (1985a). Embryonic development of the rat cerebellum. I. Delineation of the cerebellar primordium and early cell movements. J. Comp. Neurol. 231, 1–26. 10.1002/cne.902310103 3968224

[B5] Altman J. Bayer S. A. (1985b). Embryonic development of the rat cerebellum. II. Translocation and regional distribution of the deep neurons. J. Comp. Neurol. 231, 27–41. 10.1002/cne.902310104 3968227

[B6] Altman J. Bayer S. A. (1985c). Embryonic development of the rat cerebellum. III. Regional differences in the time of origin, migration and settling of Purkinje cells. J. Comp. Neurol. 231, 42–65. 10.1002/cne.902310105 3968228

[B7] Altman J. Bayer S. A. (1997). Development of the Cerebellar System: In Relation to its Evolution, Structure and Functions. FLorida: CRC Press.

[B8] Alvarez Otero R. Sotelo C. Alvarado-Mallart R. M. (1993). Chick/quail chimeras with partial cerebellar grafts: an analysis of the origin and migration of cerebellar cells. J. Comp. Neurol. 333, 597–615. 10.1002/cne.903330411 7690372

[B9] Ambrosi G. Flace P. Lorusso L. Girolamo F. Rizzi A. Bosco L.. (2007). Non-traditional large neurons in the granular layer of the cerebellar cortex. Eur. J. Histochem. 51(Suppl. 1), 59–64. 17703595

[B10] Amir R. E. Van den Veyver I. B. Wan M. Tran C. Q. Francke U. Zoghbi H. Y. (1999). Rett syndrome is caused by mutations in X-linked MECP2, encoding methyl-CpG-binding protein 2. Nat. Genet. 23, 185–188. 10.1038/13810 10508514

[B11] Anttonen A. K. Mahjneh I. Hämäläinen R. H. Lagier-Tourenne C. Kopra O. Waris L.. (2005). The gene disrupted in Marinesco-Sjögren syndrome encodes SIL1, an HSPA5 cochaperone. Nat. Genet. 37, 1309–1311. 10.1038/ng1677 16282978

[B12] Armstrong C. L. Duffin C. A. McFarland R. Vogel M. W. (2011). Mechanisms of compartmental purkinje cell death and survival in the lurcher mutant mouse. Cerebellum 10, 504–514. 10.1007/s12311-010-0231-4 21104177

[B13] Artavanis-Tsakonas S. Rand M. D. Lake R. J. (1999). Notch signaling: cell fate control and signal integration in development. Science 284, 770–776. 10.1126/science.284.5415.770 10221902

[B14] Aspatwar A. Tolvanen M. E. Jokitalo E. Parikka M. Ortutay C. Harjula S. K.. (2013). Abnormal cerebellar development and ataxia in CARP VIII morphant zebrafish. Hum. Mol. Genet. 22, 417–432. 10.1093/hmg/dds438 23087022

[B15] Azevedo F. A. Carvalho L. R. Grinberg L. T. Farfel J. M. Ferretti R. E. Leite R. E.. (2009). Equal numbers of neuronal and nonneuronal cells make the human brain an isometrically scaled-up primate brain. J. Comp. Neurol. 513, 532–541. 10.1002/cne.21974 19226510

[B16] Bailey K. Rahimi Balaei M. Mannan A. Del Bigio M. R. Marzban H. (2014). Purkinje cell compartmentation in the cerebellum of the lysosomal Acid phosphatase 2 mutant mouse (nax—naked-ataxia mutant mouse). PLoS One 9:e94327. 10.1371/journal.pone.0094327 24722417 PMC3983142

[B17] Bailey K. Rahimi Balaei M. Mehdizadeh M. Marzban H. (2013). Spatial and temporal expression of lysosomal acid phosphatase 2 (ACP2) reveals dynamic patterning of the mouse cerebellar cortex. Cerebellum 12, 870–881. 10.1007/s12311-013-0502-y 23780826

[B18] Bak M. Silahtaroglu A. Moller M. Christensen M. Rath M. F. Skryabin B.. (2008). MicroRNA expression in the adult mouse central nervous system. RNA 14, 432–444. 10.1261/rna.783108 18230762 PMC2248253

[B19] Barber B. A. Liyanage V. R. Zachariah R. M. Olson C. O. Bailey M. A. Rastegar M. (2013). Dynamic expression of MEIS1 homeoprotein in E14.5 forebrain and differentiated forebrain-derived neural stem cells. Ann. Anat. 195, 431–440. 10.1016/j.aanat.2013.04.005 23756022

[B20] Barber B. A. Rastegar M. (2010). Epigenetic control of Hox genes during neurogenesis, development and disease. Ann. Anat. 192, 261–274. 10.1016/j.aanat.2010.07.009 20739155

[B21] Basille M. Cartier D. Vaudry D. Lihrmann I. Fournier A. Freger P.. (2006). Localization and characterization of pituitary adenylate cyclase-activating polypeptide receptors in the human cerebellum during development. J. Comp. Neurol. 496, 468–478. 10.1002/cne.20934 16572459

[B22] Basson M. A. (2014). Epistatic interactions between Chd7 and Fgf8 during cerebellar development: implications for CHARGE syndrome. Rare Dis. 2:e28688. 10.4161/rdis.28688 25054096 PMC4091603

[B23] Bell C. C. (2002). Evolution of cerebellum-like structures. Brain Behav. Evol. 59, 312–326. 10.1159/000063567 12207086

[B24] Bell J. E. Sandison A. Boddy J. Franks A. J. Batcup G. Calvert R.. (1989). Development of the cerebellum with particular reference to cellular differentiation in the external granular layer. Early Hum. Dev. 19, 199–211. 10.1016/0378-3782(89)90080-7 2505998

[B25] Bellamy T. C. (2006). Interactions between Purkinje neurones and Bergmann glia. Cerebellum 5, 116–126. 10.1080/14734220600724569 16818386

[B26] Ben-Arie N. Bellen H. J. Armstrong D. L. McCall A. E. Gordadze P. R. Guo Q.. (1997). Math1 is essential for genesis of cerebellar granule neurons. Nature 390, 169–172. 10.1038/36579 9367153

[B27] Beres T. M. Masui T. Swift G. H. Shi L. Henke R. M. Macdonald R. J. (2006). PTF1 is an organ-specific and Notch-independent basic helix-loop-helix complex containing the mammalian Suppressor of Hairless (RBP-J) or its paralogue, RBP-L. Mol. Cell. Biol. 26, 117–130. 10.1128/mcb.26.1.117-130.2006 16354684 PMC1317634

[B28] Blaess S. Corrales J. D. Joyner A. L. (2006). Sonic hedgehog regulates Gli activator and repressor functions with spatial and temporal precision in the mid/hindbrain region. Development 133, 1799–1809. 10.1242/dev.02339 16571630

[B29] Blank M. C. Grinberg I. Aryee E. Laliberte C. Chizhikov V. V. Henkelman R. M.. (2011). Multiple developmental programs are altered by loss of Zic1 and Zic4 to cause Dandy-Walker malformation cerebellar pathogenesis. Development 138, 1207–1216. 10.1242/dev.054114 21307096 PMC3042874

[B30] Bogaert M. G. Belpaire F. M. (1977). Studies of papaverine metabolism in animals. Verh. K. Acad. Geneeskd. Belg. 39, 65–103. 337719

[B31] Boukhtouche F. Doulazmi M. Frederic F. Dusart I. Brugg B. Mariani J. (2006). RORalpha, a pivotal nuclear receptor for Purkinje neuron survival and differentiation: from development to ageing. Cerebellum 5, 97–104. 10.1080/14734220600750184 16818384

[B32] Bulfone A. Martinez S. Marigo V. Campanella M. Basile A. Quaderi N.. (1999). Expression pattern of the Tbr2 (Eomesodermin) gene during mouse and chick brain development. Mech. Dev. 84, 133–138. 10.1016/s0925-4773(99)00053-2 10473127

[B33] Bulfone A. Smiga S. M. Shimamura K. Peterson A. Puelles L. Rubenstein J. L. (1995). T-brain-1: a homolog of Brachyury whose expression defines molecularly distinct domains within the cerebral cortex. Neuron 15, 63–78. 10.1016/0896-6273(95)90065-9 7619531

[B34] Butler A. B. Hodos W. (2005). Comparative Vertebrate Neuroanatomy: Evolution and Adaptation. 2nd Edn. New York: Wiley.

[B35] Butts T. Green M. J. Wingate R. J. (2014). Development of the cerebellum: simple steps to make a ‘little brain’. Development 141, 4031–4041. 10.1242/dev.106559 25336734

[B36] Cabrera O. Dougherty J. Singh S. Swiney B. S. Farber N. B. Noguchi K. K. (2014). Lithium protects against glucocorticoid induced neural progenitor cell apoptosis in the developing cerebellum. Brain Res. 1545, 54–63. 10.1016/j.brainres.2013.12.014 24361977 PMC3919041

[B37] Caddy K. W. Biscoe T. J. (1979). Structural and quantitative studies on the normal C3H and Lurcher mutant mouse. Philos. Trans. R. Soc. Lond. B Biol. Sci. 287, 167–201. 10.1098/rstb.1979.0055 41272

[B38] Campbell N. C. Armstrong D. M. (1983). Topographical localization in the olivocerebellar projection in the rat: an autoradiographic study. Brain Res. 275, 235–249. 10.1016/0006-8993(83)90985-x 6194853

[B39] Carletti B. Rossi F. (2008). Neurogenesis in the cerebellum. Neuroscientist 14, 91–100. 10.1177/1073858407304629 17911211

[B40] Carthew R. W. Sontheimer E. J. (2009). Origins and mechanisms of miRNAs and siRNAs. Cell 136, 642–655. 10.1016/j.cell.2009.01.035 19239886 PMC2675692

[B41] Castejón O. J. (2013). Confocal laser scanning microscopy and immunohistochemistry of cerebellar Lugaro cells. Biocell 37, 29–36. 24392579

[B42] Catsicas S. Thanos S. Clarke P. (1987). Major role for neuronal death during brain development: refinement of topographical connections. Proc. Natl. Acad. Sci. U S A 84, 8165–8168. 10.1073/pnas.84.22.8165 3479784 PMC299499

[B43] Cecconi F. Levine B. (2008). The role of autophagy in mammalian development: cell makeover rather than cell death. Dev. Cell 15, 344–357. 10.1016/j.devcel.2008.08.012 18804433 PMC2688784

[B44] Chan W. Y. Lorke D. E. Tiu S. C. Yew D. T. (2002). Proliferation and apoptosis in the developing human neocortex. Anat. Rec. 267, 261–276. 10.1002/ar.10100 12124904

[B45] Chédotal A. (2010). Should I stay or should I go? Becoming a granule cell. Trends Neurosci. 33, 163–172. 10.1016/j.tins.2010.01.004 20138673

[B46] Cheng X. S. Li M. S. Du J. Jiang Q. Y. Wang L. Yan S. Y.. (2011). Neuronal apoptosis in the developing cerebellum. Anat. Histol. Embryol. 40, 21–27. 10.1111/j.1439-0264.2010.01033.x 21231956

[B48] Chizhikov V. V. Lindgren A. G. Currle D. S. Rose M. F. Monuki E. S. Millen K. J. (2006a). The roof plate regulates cerebellar cell-type specification and proliferation. Development 133, 2793–2804. 10.1242/dev.02441 16790481

[B47] Chizhikov V. Steshina E. Roberts R. Ilkin Y. Washburn L. Millen K. J. (2006b). Molecular definition of an allelic series of mutations disrupting the mouse Lmx1a (dreher) gene. Mamm. Genome 17, 1025–1032. 10.1007/s00335-006-0033-7 17019651

[B49] Cho K. H. Rodríguez-Vázquez J. F. Kim J. H. Abe H. Murakami G. Cho B. H. (2011). Early fetal development of the human cerebellum. Surg. Radiol. Anat. 33, 523–530. 10.1007/s00276-011-0796-8 21380713

[B50] Choi B. H. Lapham L. W. (1980). Evolution of Bergmann glia in developing human fetal cerebellum: a Golgi, electron microscopic and immunofluorescent study. Brain Res. 190, 369–383. 10.1016/0006-8993(80)90280-2 6989450

[B51] Chung S. H. Marzban H. Aldinger K. Dixit R. Millen K. Schuurmans C.. (2011). Zac1 plays a key role in the development of specific neuronal subsets in the mouse cerebellum. Neural Dev. 6:25. 10.1186/1749-8104-6-25 21592321 PMC3113315

[B52] Chung S. H. Marzban H. Hawkes R. (2009). Compartmentation of the cerebellar nuclei of the mouse. Neuroscience 161, 123–138. 10.1016/j.neuroscience.2009.03.037 19306913

[B53] Cobb S. Guy J. Bird A. (2010). Reversibility of functional deficits in experimental models of Rett syndrome. Biochem. Soc. Trans. 38, 498–506. 10.1042/BST0380498 20298210

[B54] Corrales J. D. Rocco G. L. Blaess S. Guo Q. Joyner A. L. (2004). Spatial pattern of sonic hedgehog signaling through Gli genes during cerebellum development. Development 131, 5581–5590. 10.1242/dev.01438 15496441

[B55] Cotterill R. M. (2001). Cooperation of the basal ganglia, cerebellum, sensory cerebrum and hippocampus: possible implications for cognition, consciousness, intelligence and creativity. Prog. Neurobiol. 64, 1–33. 10.1016/s0301-0082(00)00058-7 11250060

[B56] Courchesne E. (1997). Brainstem, cerebellar and limbic neuroanatomical abnormalities in autism. Curr. Opin. Neurobiol. 7, 269–278. 10.1016/S0959-4388(97)80016-5 9142760

[B57] Courchesne E. Yeung-Courchesne R. Press G. A. Hesselink J. R. Jernigan T. L. (1988). Hypoplasia of cerebellar vermal lobules VI and VII in autism. N. Engl. J. Med. 318, 1349–1354. 10.1056/nejm198805263182102 3367935

[B58] Crespo-Enriquez I. Partanen J. Martinez S. Echevarria D. (2012). Fgf8-related secondary organizers exert different polarizing planar instructions along the mouse anterior neural tube. PLoS One 7:e39977. 10.1371/journal.pone.0039977 22792203 PMC3391221

[B60] Danielian P. S. McMahon A. P. (1996). Engrailed-1 as a target of the Wnt-1 signalling pathway in vertebrate midbrain development. Nature 383, 332–334. 10.1038/383332a0 8848044

[B61] Darnell J. C. Klann E. (2013). The translation of translational control by FMRP: therapeutic targets for FXS. Nat. Neurosci. 16, 1530–1536. 10.1038/nn.3379 23584741 PMC3999698

[B64] Del Cerro M. Swarz J. R. (1976). Prenatal development of Bergmann glial fibres in rodent cerebellum. J. Neurocytol. 5, 669–676. 10.1007/bf01181580 1003259

[B65] Delcuve G. P. Rastegar M. Davie J. R. (2009). Epigenetic control. J. Cell. Physiol. 219, 243–250. 10.1002/jcp.21678 19127539

[B66] Delong G. R. (2005). “The cerebellum in autism,” in The Neurology of Autism, ed Coleman M. (New York), 75–90.

[B62] de Luca A. Weller M. Fontana A. (1996). TGF-beta-induced apoptosis of cerebellar granule neurons is prevented by depolarization. J. Neurosci. 16, 4174–4185. 8753879 10.1523/JNEUROSCI.16-13-04174.1996 PMC6578986

[B67] Denzel A. Molinari M. Trigueros C. Martin J. E. Velmurgan S. Brown S.. (2002). Early postnatal death and motor disorders in mice congenitally deficient in calnexin expression. Mol. Cell. Biol. 22, 7398–7404. 10.1128/mcb.22.21.7398-7404.2002 12370287 PMC135653

[B68] Deo K. Bijlani V. Deo M. G. (1979). “Physiological” and cytotoxic cell death in protein deficiency. A study in developing cerebellum in rats. Acta Neuropathol. 46, 221–225. 10.1007/bf00690848 572615

[B69] Devor A. (2000). Is the cerebellum like cerebellar-like structures? Brain Res. Brain Res. Rev. 34, 149–156. 10.1016/s0165-0173(00)00045-x 11113505

[B63] De Zeeuw C. I. Berrebi A. S. (1995). Postsynaptic targets of Purkinje cell terminals in the cerebellar and vestibular nuclei of the rat. Eur. J. Neurosci. 7, 2322–2333. 10.1111/j.1460-9568.1995.tb00653.x 8563981

[B70] Di Bartolomeo S. Nazio F. Cecconi F. (2010). The role of autophagy during development in higher eukaryotes. Traffic 11, 1280–1289. 10.1111/j.1600-0854.2010.01103.x 20633243

[B71] DiCicco-Bloom E. Lord C. Zwaigenbaum L. Courchesne E. Dager S. R. Schmitz C.. (2006). The developmental neurobiology of autism spectrum disorder. J. Neurosci. 26, 6897–6906. 10.1523/JNEUROSCI.1712-06.2006 16807320 PMC6673916

[B59] D’Mello S. R. Galli C. Ciotti T. Calissano P. (1993). Induction of apoptosis in cerebellar granule neurons by low potassium: inhibition of death by insulin-like growth factor I and cAMP. Proc. Natl. Acad. Sci. U S A 90, 10989–10993. 10.1073/pnas.90.23.10989 8248201 PMC47907

[B72] Du X. Wang J. Zhu H. Rinaldo L. Lamar K. M. Palmenberg A. C.. (2013). Second cistron in CACNA1A gene encodes a transcription factor mediating cerebellar development and SCA6. Cell 154, 118–133. 10.1016/j.cell.2013.05.059 23827678 PMC3939801

[B73] Dun X. P. (2012). Origin of climbing fiber neurons and the definition of rhombic lip. Int. J. Dev. Neurosci. 30, 391–395. 10.1016/j.ijdevneu.2012.02.002 22406199

[B74] Ellis R. S. (1920). Norms for some structural changes in the human cerebellum from birth to old age. J. Comp. Neurol. 32, 1–33 10.1002/cne.900320102

[B75] Engelkamp D. Rashbass P. Seawright A. van Heyningen V. (1999). Role of Pax6 in development of the cerebellar system. Development 126, 3585–3596. 10409504 10.1242/dev.126.16.3585

[B76] Englund C. Kowalczyk T. Daza R. A. Dagan A. Lau C. Rose M. F.. (2006). Unipolar brush cells of the cerebellum are produced in the rhombic lip and migrate through developing white matter. J. Neurosci. 26, 9184–9195. 10.1523/jneurosci.1610-06.2006 16957075 PMC6674506

[B77] Eulalio A. Mano M. Dal Ferro M. Zentilin L. Sinagra G. Zacchigna S.. (2012). Functional screening identifies miRNAs inducing cardiac regeneration. Nature 492, 376–381. 10.1038/nature11739 23222520

[B78] Ezeonwuka C. Rastegar M. (2014). MeCP2-related diseases and animal models. Diseases 2, 45–70 10.3390/diseases2010045 PMC5298922 28191346

[B79] Fatemi S. H. Aldinger K. A. Ashwood P. Bauman M. L. Blaha C. D. Blatt G. J.. (2012). Consensus paper: pathological role of the cerebellum in autism. Cerebellum 11, 777–807. 10.1007/s12311-012-0355-9 22370873 PMC3677555

[B80] Finckbone V. Oomman S. K. Strahlendorf H. K. Strahlendorf J. C. (2009). Regional differences in the temporal expression of non-apoptotic caspase-3-positive bergmann glial cells in the developing rat cerebellum. Front. Neuroanat. 3:3. 10.3389/neuro.05.003.2009 19503747 PMC2691149

[B81] Fink A. J. Englund C. Daza R. A. Pham D. Lau C. Nivison M.. (2006). Development of the deep cerebellar nuclei: transcription factors and cell migration from the rhombic lip. J. Neurosci. 26, 3066–3076. 10.1523/jneurosci.5203-05.2006 16540585 PMC6673970

[B82] Follert P. Cremer H. Beclin C. (2014). MicroRNAs in brain development and function: a matter of flexibility and stability. Front. Mol. Neurosci. 7:5. 10.3389/fnmol.2014.00005 24570654 PMC3916726

[B83] Friede R. L. (1973). Dating the development of human cerebellum. Acta Neuropathol. 23, 48–58. 10.1007/bf00689004 4698523

[B84] Furuichi T. Shiraishi-Yamaguchi Y. Sato A. Sadakata T. Huang J. Shinoda Y.. (2011). Systematizing and cloning of genes involved in the cerebellar cortex circuit development. Neurochem. Res. 36, 1241–1252. 10.1007/s11064-011-0398-1 21243430

[B85] Gadson D. R. Emery J. L. (1976). Some quantitative morphological aspects of post-natal human cerebellar growth. J. Neurol. Sci. 29, 137–148. 10.1016/0022-510x(76)90166-0 978206

[B86] Gajjar A. J. Robinson G. W. (2014). Medulloblastoma-translating discoveries from the bench to the bedside. Nat. Rev. Clin. Oncol. 11, 714–722. 10.1038/nrclinonc.2014.181 25348790

[B87] Gao J. H. Parsons L. M. Bower J. M. Xiong J. Li J. Fox P. T. (1996). Cerebellum implicated in sensory acquisition and discrimination rather than motor control. Science 272, 545–547. 10.1126/science.272.5261.545 8614803

[B88] Garel C. Fallet-Bianco C. Guibaud L. (2011). The fetal cerebellum: development and common malformations. J. Child Neurol. 26, 1483–1492. 10.1177/0883073811420148 21954430

[B89] Gelpi E. Budka H. Preusser M. (2013). External granular cell layer bobbling: a distinct histomorphological feature of the developing human cerebellum. Clin. Neuropathol. 32, 42–50. 10.5414/np300518 22943957

[B90] Giacometti E. Luikenhuis S. Beard C. Jaenisch R. (2007). Partial rescue of MeCP2 deficiency by postnatal activation of MeCP2. Proc. Natl. Acad. Sci. U S A 104, 1931–1936. 10.1073/pnas.0610593104 17267601 PMC1794312

[B91] Gibbons R. J. Higgs D. R. (2000). Molecular-clinical spectrum of the ATR-X syndrome. Am. J. Med. Genet. 97, 204–212. 10.1002/1096-8628(200023)97:3<204::AID-AJMG1038>3.0.CO;2-X 11449489

[B92] Glickstein M. (1993). Motor skills but not cognitive tasks. Trends Neurosci. 16, 450–451; discussion 453–454. 10.1016/0166-2236(93)90074-V 7507616

[B93] Glickstein M. Strata P. Voogd J. (2009). Cerebellum: history. Neuroscience 162, 549–559. 10.1016/j.neuroscience.2009.02.054 19272426

[B94] Gold D. A. Gent P. M. Hamilton B. A. (2007). ROR alpha in genetic control of cerebellum development: 50 staggering years. Brain Res. 1140, 19–25. 10.1016/j.brainres.2005.11.080 16427031

[B95] Goldowitz D. Cushing R. C. Laywell E. D’arcangelo G. Sheldon M. Sweet H. O.. (1997). Cerebellar disorganization characteristic of reeler in scrambler mutant mice despite presence of reelin. J. Neurosci. 17, 8767–8777. 9348346 10.1523/JNEUROSCI.17-22-08767.1997 PMC6573071

[B96] Goldowitz D. Hamre K. (1998). The cells and molecules that make a cerebellum. Trends Neurosci. 21, 375–382. 10.1016/s0166-2236(98)01313-7 9735945

[B97] Greco C. M. Berman R. F. Martin R. M. Tassone F. Schwartz P. H. Chang A.. (2006). Neuropathology of fragile X-associated tremor/ataxia syndrome (FXTAS). Brain 129, 243–255. 10.1093/brain/awh683 16332642

[B98] Griffin W. S. Woodward D. J. Chanda R. (1978). Quantification of cell death in developing cerebellum by a 14C tracer method. Brain Res. Bull. 3, 369–372. 10.1016/0361-9230(78)90105-3 318207

[B99] Gudovic R. Milutinovic B. Ristanovic D. (1998). Dynamics of granule cells migration into the internal granular layer in developing human cerebellum. J. Hirnforsch. 39, 223–229. 10022346

[B100] Guibinga G. H. Hrustanovic G. Bouic K. Jinnah H. A. Friedmann T. (2012). MicroRNA-mediated dysregulation of neural developmental genes in HPRT deficiency: clues for Lesch-Nyhan disease? Hum. Mol. Genet. 21, 609–622. 10.1093/hmg/ddr495 22042773 PMC3259014

[B101] Guihard-Costa A. M. Larroche J. C. (1990). Differential growth between the fetal brain and its infratentorial part. Early Hum. Dev. 23, 27–40. 10.1016/0378-3782(90)90126-4 2209474

[B102] Guillemot F. Molnár Z. Tarabykin V. Stoykova A. (2006). Molecular mechanisms of cortical differentiation. Eur. J. Neurosci. 23, 857–868. 10.1111/j.1460-9568.2006.04626.x 16519651

[B103] Hagerman R. J. Leehey M. Heinrichs W. Tassone F. Wilson R. Hills J.. (2001). Intention tremor, parkinsonism and generalized brain atrophy in male carriers of fragile X. Neurology 57, 127–130. 10.1212/wnl.57.1.127 11445641

[B104] Haldipur P. Bharti U. Alberti C. Sarkar C. Gulati G. Iyengar S.. (2011). Preterm delivery disrupts the developmental program of the cerebellum. PLoS One 6:e23449. 10.1371/journal.pone.0023449 21858122 PMC3157376

[B105] Haldipur P. Bharti U. Govindan S. Sarkar C. Iyengar S. Gressens P.. (2012). Expression of Sonic hedgehog during cell proliferation in the human cerebellum. Stem Cells Dev. 21, 1059–1068. 10.1089/scd.2011.0206 21732818

[B106] Hallahan B. Daly E. M. McAlonan G. Loth E. Toal F. O’Brien F.. (2009). Brain morphometry volume in autistic spectrum disorder: a magnetic resonance imaging study of adults. Psychol. Med. 39, 337–346. 10.1017/s0033291708003383 18775096

[B107] Hallonet M. Alvarado-Mallart R. M. (1997). The chick/quail chimeric system: a model for early cerebellar development. Perspect. Dev. Neurobiol. 5, 17–31. 9509515

[B108] Hara T. Nakamura K. Matsui M. Yamamoto A. Nakahara Y. Suzuki-Migishima R.. (2006). Suppression of basal autophagy in neural cells causes neurodegenerative disease in mice. Nature 441, 885–889. 10.1038/nature04724 16625204

[B110] Hashimoto M. Hibi M. (2012). Development and evolution of cerebellar neural circuits. Dev. Growth Differ. 54, 373–389. 10.1111/j.1440-169x.2012.01348.x 22524607

[B109] Hashimoto K. Kano M. (2013). Synapse elimination in the developing cerebellum. Cell. Mol. Life Sci. 70, 4667–4680. 10.1007/s00018-013-1405-2 23811844 PMC3830199

[B111] Hashimoto M. Mikoshiba K. (2003). Mediolateral compartmentalization of the cerebellum is determined on the “birth date” of Purkinje cells. J. Neurosci. 23, 11342–11351. 14672998 10.1523/JNEUROSCI.23-36-11342.2003 PMC6740522

[B112] Hatten M. E. Heintz N. (1995). Mechanisms of neural patterning and specification in the developing cerebellum. Annu. Rev. Neurosci. 18, 385–408. 10.1146/annurev.neuro.18.1.385 7605067

[B113] Heikinheimo M. Lawshé A. Shackleford G. M. Wilson D. B. MacArthur C. A. (1994). Fgf-8 expression in the post-gastrulation mouse suggests roles in the development of the face, limbs and central nervous system. Mech. Dev. 48, 129–138. 10.1016/0925-4773(94)90022-1 7873403

[B114] Heinsen H. (1977). Quantitative anatomical studies on the postnatal development of the cerebellum of the albino rat. Anat. Embryol. (Berl) 151, 201–218. 10.1007/bf00297481 920968

[B115] Heinsen H. (1978). Postnatal quantitative changes in the cerebellar uvula of albino rats. Anat. Embryol. (Berl) 154, 285–304. 10.1007/bf00345658 707819

[B116] Herrup K. Wilczynski S. L. (1982). Cerebellar cell degeneration in the leaner mutant mouse. Neuroscience 7, 2185–2196. 10.1016/0306-4522(82)90129-4 7145091

[B117] Hevner R. F. (2006). From radial glia to pyramidal-projection neuron: transcription factor cascades in cerebral cortex development. Mol. Neurobiol. 33, 33–50. 10.1385/mn:33:1:033 16388109

[B118] Hevner R. F. Hodge R. D. Daza R. A. Englund C. (2006). Transcription factors in glutamatergic neurogenesis: conserved programs in neocortex, cerebellum and adult hippocampus. Neurosci. Res. 55, 223–233. 10.1016/j.neures.2006.03.004 16621079

[B119] Hibi M. Shimizu T. (2012). Development of the cerebellum and cerebellar neural circuits. Dev. Neurobiol. 72, 282–301. 10.1002/dneu.20875 21309081

[B120] Hidalgo-Sánchez M. Millet S. Bloch-Gallego E. Alvarado-Mallart R. M. (2005). Specification of the meso-isthmo-cerebellar region: the Otx2/Gbx2 boundary. Brain Res. Brain Res. Rev. 49, 134–149. 10.1016/j.brainresrev.2005.01.010 16111544

[B121] Hiraoka Y. Komine O. Nagaoka M. Bai N. Hozumi K. Tanaka K. (2013). Delta-like 1 regulates Bergmann glial monolayer formation during cerebellar development. Mol. Brain 6:25. 10.1186/1756-6606-6-25 23688253 PMC3724498

[B122] Hohjoh H. Fukushima T. (2007). Expression profile analysis of microRNA (miRNA) in mouse central nervous system using a new miRNA detection system that examines hybridization signals at every step of washing. Gene 391, 39–44. 10.1016/j.gene.2006.11.018 17229533

[B123] Hoshino M. (2006a). Molecular machinery governing GABAergic neuron specification in the cerebellum. Cerebellum 5, 193–198. 10.1080/14734220600589202 16997750

[B124] Hoshino M. (2006b). Molecular mechanisms underlying glutamatergic vs. GABAergic neuronal subtype specification in the cerebellum. Seikagaku 78, 130–132. 16541804

[B125] Hoshino M. (2012). Neuronal subtype specification in the cerebellum and dorsal hindbrain. Dev. Growth Differ. 54, 317–326. 10.1111/j.1440-169x.2012.01330.x 22404503

[B126] Hoshino M. Nakamura S. Mori K. Kawauchi T. Terao M. Nishimura Y. V.. (2005). Ptf1a, a bHLH transcriptional gene, defines GABAergic neuronal fates in cerebellum. Neuron 47, 201–213. 10.1016/j.neuron.2005.06.007 16039563

[B127] Houtmeyers R. Souopgui J. Tejpar S. Arkell R. (2013). The ZIC gene family encodes multi-functional proteins essential for patterning and morphogenesis. Cell. Mol. Life Sci. 70, 3791–3811. 10.1007/s00018-013-1285-5 23443491 PMC11113920

[B128] Huang G. J. Edwards A. Tsai C. Y. Lee Y. S. Peng L. Era T.. (2014). Ectopic cerebellar cell migration causes maldevelopment of Purkinje cells and abnormal motor behaviour in Cxcr4 null mice. PLoS One 9:e86471. 10.1371/journal.pone.0086471 24516532 PMC3917845

[B129] Huang H. Rastegar M. Bodner C. Goh S. L. Rambaldi I. Featherstone M. (2005). MEIS C termini harbor transcriptional activation domains that respond to cell signaling. J. Biol. Chem. 280, 10119–10127. 10.1074/jbc.m413963200 15654074

[B130] Ieraci A. Forni P. E. Ponzetto C. (2002). Viable hypomorphic signaling mutant of the Met receptor reveals a role for hepatocyte growth factor in postnatal cerebellar development. Proc. Natl. Acad. Sci. U S A 99, 15200–15205. 10.1073/pnas.222362099 12397180 PMC137567

[B131] Impagnatiello F. Guidotti A. R. Pesold C. Dwivedi Y. Caruncho H. Pisu M. G.. (1998). A decrease of reelin expression as a putative vulnerability factor in schizophrenia. Proc. Natl. Acad. Sci. U S A 95, 15718–15723. 10.1073/pnas.95.26.15718 9861036 PMC28110

[B132] Itasaki N. Nakamura H. (1992). Rostrocaudal polarity of the tectum in birds: correlation of en gradient and topographic order in retinotectal projection. Neuron 8, 787–798. 10.1016/0896-6273(92)90099-y 1348950

[B133] Ito M. (1984). The modifiable neuronal network of the cerebellum. Jpn. J. Physiol. 34, 781–792. 10.2170/jjphysiol.34.781 6099855

[B134] Jaarsma D. Ruigrok T. J. Caffé R. Cozzari C. Levey A. I. Mugnaini E.. (1997). Cholinergic innervation and receptors in the cerebellum. Prog. Brain Res. 114, 67–96. 10.1016/s0079-6123(08)63359-2 9193139

[B135] James S. J. Shpyleva S. Melnyk S. Pavliv O. Pogribny I. P. (2014). Elevated 5-hydroxymethylcytosine in the Engrailed-2 (EN-2) promoter is associated with increased gene expression and decreased MeCP2 binding in autism cerebellum. Transl. Psychiatry 4:e460. 10.1038/tp.2014.87 25290267 PMC4350522

[B137] Ji Z. Hawkes R. (1994). Topography of Purkinje cell compartments and mossy fiber terminal fields in lobules II and III of the rat cerebellar cortex: spinocerebellar and cuneocerebellar projections. Neuroscience 61, 935–954. 10.1016/0306-4522(94)90414-6 7530818

[B138] Jiang Y. Kumada T. Cameron D. B. Komuro H. (2008). Cerebellar granule cell migration and the effects of alcohol. Dev. Neurosci. 30, 7–23. 10.1159/000109847 18075250

[B139] Johnson E. M. Jr. Deckwerth T. L. (1993). Molecular mechanisms of developmental neuronal death. Annu. Rev. Neurosci. 16, 31–46. 10.1146/annurev.neuro.16.1.31 8460896

[B140] Kajiura S. M. Cornett A. D. Yopak K. E. (2010). Sensory Adaptations to the Environment: Electroreceptors as a Case Study. New York: CRC Press.

[B141] Kalinichenko S. G. Okhotin V. E. (2005). Unipolar brush cells—a new type of excitatory interneuron in the cerebellar cortex and cochlear nuclei of the brainstem. Neurosci. Behav. Physiol. 35, 21–36. 10.1023/b:neab.0000049648.20702.ad 15739785

[B142] Kalyani A. Hobson K. Rao M. S. (1997). Neuroepithelial stem cells from the embryonic spinal cord: isolation, characterization and clonal analysis. Dev. Biol. 186, 202–223. 10.1006/dbio.1997.8592 9205140

[B143] Kapur R. P. Mahony B. S. Finch L. Siebert J. R. (2009). Normal and abnormal anatomy of the cerebellar vermis in midgestational human fetuses. Birth Defects Res. A Clin. Mol. Teratol. 85, 700–709. 10.1002/bdra.20589 19441098

[B136] Kaslin J. Brand M. (2013). Cerebellar Development and Neurogenesis in Zebrafish. Netherlands: Springer.

[B144] Katahira T. Sato T. Sugiyama S. Okafuji T. Araki I. Funahashi J.. (2000). Interaction between Otx2 and Gbx2 defines the organizing center for the optic tectum. Mech. Dev. 91, 43–52. 10.1016/s0925-4773(99)00262-2 10704829

[B145] Kawauchi T. Chihama K. Nishimura Y. V. Nabeshima Y. Hoshino M. (2005). MAP1B phosphorylation is differentially regulated by Cdk5/p35, Cdk5/p25 and JNK. Biochem. Biophys. Res. Commun. 331, 50–55. 10.1016/j.bbrc.2005.03.132 15845356

[B146] Kim J. Y. Marzban H. Chung S. H. Watanabe M. Eisenman L. M. Hawkes R. (2009). Purkinje cell compartmentation of the cerebellum of microchiropteran bats. J. Comp. Neurol. 517, 193–209. 10.1002/cne.22147 19731335

[B147] Kitao Y. Hashimoto K. Matsuyama T. Iso H. Tamatani T. Hori O.. (2004). ORP150/HSP12A regulates Purkinje cell survival: a role for endoplasmic reticulum stress in cerebellar development. J. Neurosci. 24, 1486–1496. 10.1523/jneurosci.4029-03.2004 14960622 PMC6730325

[B148] Klionsky D. J. (2005). The molecular machinery of autophagy: unanswered questions. J. Cell Sci. 118, 7–18. 10.1242/jcs.01620 15615779 PMC1828869

[B149] Kobrossy L. Rastegar M. Featherstone M. (2006). Interplay between chromatin and trans-acting factors regulating the Hoxd4 promoter during neural differentiation. J. Biol. Chem. 281, 25926–25939. 10.1074/jbc.m602555200 16757478

[B150] Komatsu M. Waguri S. Chiba T. Murata S. Iwata J.-I. Tanida I.. (2006). Loss of autophagy in the central nervous system causes neurodegeneration in mice. Nature 441, 880–884. 10.1038/nature04723 16625205

[B151] Komatsu M. Waguri S. Koike M. Sou Y.-S. Ueno T. Hara T.. (2007a). Homeostatic levels of p62 control cytoplasmic inclusion body formation in autophagy-deficient mice. Cell 131, 1149–1163. 10.1016/j.cell.2007.10.035 18083104

[B152] Komatsu M. Wang Q. J. Holstein G. R. Friedrich V. L. Iwata J.-I. Kominami E.. (2007b). Essential role for autophagy protein Atg7 in the maintenance of axonal homeostasis and the prevention of axonal degeneration. Proc. Natl. Acad. Sci. U S A 104, 14489–14494. 10.1073/pnas.0701311104 17726112 PMC1964831

[B153] Komuro H. Rakic P. (1996). Intracellular Ca2+ fluctuations modulate the rate of neuronal migration. Neuron 17, 275–285. 10.1016/s0896-6273(00)80159-2 8780651

[B154] Komuro H. Yacubova E. (2003). Recent advances in cerebellar granule cell migration. Cell. Mol. Life Sci. 60, 1084–1098. 12861377 10.1007/s00018-003-2248-z PMC11138937

[B155] Krapp A. Knöfler M. Ledermann B. Bürki K. Berney C. Zoerkler N.. (1998). The bHLH protein PTF1–p48 is essential for the formation of the exocrine and the correct spatial organization of the endocrine pancreas. Genes Dev. 12, 3752–3763. 10.1101/gad.12.23.3752 9851981 PMC317250

[B156] Kriaucionis S. Bird A. (2004). The major form of MeCP2 has a novel N-terminus generated by alternative splicing. Nucleic Acids Res. 32, 1818–1823. 10.1093/nar/gkh349 15034150 PMC390342

[B157] Krueger B. K. Burne J. F. Raff M. C. (1995). Evidence for large-scale astrocyte death in the developing cerebellum. J. Neurosci. 15, 3366–3374. 7751916 10.1523/JNEUROSCI.15-05-03366.1995 PMC6578202

[B158] Kuang Y. Liu Q. Shu X. Zhang C. Huang N. Li J.. (2012). Dicer1 and MiR-9 are required for proper Notch1 signaling and the Bergmann glial phenotype in the developing mouse cerebellum. Glia 60, 1734–1746. 10.1002/glia.22392 22836445

[B159] Kubera C. Hernandez A. L. Heng V. Bordey A. (2012). Transient mGlu5R inhibition enhances the survival of granule cell precursors in the neonatal cerebellum. Neuroscience 219, 271–279. 10.1016/j.neuroscience.2012.05.064 22677205 PMC3402690

[B160] Kuemerle B. Gulden F. Cherosky N. Williams E. Herrup K. (2007). The mouse Engrailed genes: a window into autism. Behav. Brain Res. 176, 121–132. 10.1016/j.bbr.2006.09.009 17055592 PMC2791532

[B161] Kumada T. Lakshmana M. K. Komuro H. (2006). Reversal of neuronal migration in a mouse model of fetal alcohol syndrome by controlling second-messenger signalings. J. Neurosci. 26, 742–756. 10.1523/jneurosci.4478-05.2006 16421294 PMC6675380

[B162] Kwong W. H. Chan W. Y. Lee K. K. Fan M. Yew D. T. (2000). Neurotransmitters, neuropeptides and calcium binding proteins in developing human cerebellum: a review. Histochem. J. 32, 521–534. 10.1023/A:1004197210189 11127973

[B163] Lahuna O. Rastegar M. Maiter D. Thissen J. P. Lemaigre F. P. Rousseau G. G. (2000). Involvement of STAT5 (signal transducer and activator of transcription 5) and HNF-4 (hepatocyte nuclear factor 4) in the transcriptional control of the hnf6 gene by growth hormone. Mol. Endocrinol. 14, 285–294. 10.1210/me.14.2.285 10674400

[B164] Lainé J. Axelrad H. (1994). The candelabrum cell: a new interneuron in the cerebellar cortex. J. Comp. Neurol. 339, 159–173. 10.1002/cne.903390202 8300903

[B165] Larouche M. Beffert U. Herz J. Hawkes R. (2008). The Reelin receptors Apoer2 and Vldlr coordinate the patterning of Purkinje cell topography in the developing mouse cerebellum. PLoS One 3:e1653. 10.1371/journal.pone.0001653 18301736 PMC2242849

[B166] Larsell O. (1967). The Comparative Anatomy and Histology of the Cerebellum from Myxinoids through Birds. Minneapolis, MN: University of Minnesota Press.

[B167] Larsell O. (1970). The Comparative Anatomy and Histology of the Cerebellum from Monotremes through Apes. Minneapolis: University of Minnesota Press.

[B168] Lavezzi A. M. Ottaviani G. Terni L. Matturri L. (2006). Histological and biological developmental characterization of the human cerebellar cortex. Int. J. Dev. Neurosci. 24, 365–371. 10.1016/j.ijdevneu.2006.06.002 16893622

[B169] Lee J. W. Beebe K. Nangle L. A. Jang J. Longo-Guess C. M. Cook S. A.. (2006). Editing-defective tRNA synthetase causes protein misfolding and neurodegeneration. Nature 443, 50–55. 10.1038/nature05096 16906134

[B170] Lee K. J. Dietrich P. Jessell T. M. (2000). Genetic ablation reveals that the roof plate is essential for dorsal interneuron specification. Nature 403, 734–740. 10.1038/35001507 10693795

[B171] Leiner H. C. Leiner A. L. Dow R. S. (1991). The human cerebro-cerebellar system: its computing, cognitive and language skills. Behav. Brain Res. 44, 113–128. 10.1016/s0166-4328(05)80016-6 1751002

[B172] Lemire R. J. (1975). Normal and Abnormal Development of the Human Nervous System. Hagerstown, MD: Medical Dept., Harper and Row.

[B173] Leto K. Bartolini A. Rossi F. (2008). Development of cerebellar GABAergic interneurons: origin and shaping of the “minibrain” local connections. Cerebellum 7, 523–529. 10.1007/s12311-008-0079-z 19002744

[B174] Leto K. Rossi F. (2012). Specification and differentiation of cerebellar GABAergic neurons. Cerebellum 11, 434–435. 10.1007/s12311-011-0324-8 22090364

[B175] Leung C. Lingbeek M. Shakhova O. Liu J. Tanger E. Saremaslani P.. (2004). Bmi1 is essential for cerebellar development and is overexpressed in human medulloblastomas. Nature 428, 337–341. 10.1038/nature02385 15029199

[B176] Lewis P. D. (1975). Cell death in the germinal layers of the postnatal rat brain. Neuropathol. Appl. Neurobiol. 1, 21–29 10.1111/j.1365-2990.1975.tb00374.x

[B177] Lewis P. M. Gritli-Linde A. Smeyne R. Kottmann A. Mcmahon A. P. (2004). Sonic hedgehog signaling is required for expansion of granule neuron precursors and patterning of the mouse cerebellum. Dev. Biol. 270, 393–410. 10.1016/j.ydbio.2004.03.007 15183722

[B178] Liyanage V. Jarmasz J. Murugeshan N. Del Bigio M. Rastegar M. Davie J. (2014). DNA modifications: function and applications in normal and disease states. Biology 3, 670–723. 10.3390/biology3040670 25340699 PMC4280507

[B179] Liyanage V. R. Rastegar M. (2014). Rett syndrome and MeCP2. Neuromolecular Med. 16, 231–264. 10.1007/s12017-014-8295-9 24615633 PMC5798978

[B181] Liyanage V. R. B. Zachariah R. M. Delcuve G. P. Davie J. R. Rastegar M. (2012). “New developments in chromatin research: an epigenetic perspective,” in New Developments in Chromatin Research, eds Simpson N. M. Stewart V. J. (New York: Nova Science Publishers), 29–58.

[B180] Liyanage V. R. Zachariah R. M. Rastegar M. (2013). Decitabine alters the expression of Mecp2 isoforms via dynamic DNA methylation at the Mecp2 regulatory elements in neural stem cells. Mol. Autism 4:46. 10.1186/2040-2392-4-46 24238559 PMC3900258

[B182] Loeser J. D. Lemire R. J. Alvord E. C. Jr. (1972). The development of the folia in the human cerebellar vermis. Anat. Rec. 173, 109–113. 10.1002/ar.1091730109 5028060

[B183] Loo D. T. (2011). In situ detection of apoptosis by the TUNEL assay: an overview of techniques. Methods Mol. Biol. 682, 3–13. 10.1007/978-1-60327-409-8_1 21057916

[B184] Lopes C. Delezoide A. L. Delabar J. M. Rachidi M. (2006). BARHL1 homeogene, the human ortholog of the mouse Barhl1 involved in cerebellum development, shows regional and cellular specificities in restricted domains of developing human central nervous system. Biochem. Biophys. Res. Commun. 339, 296–304. 10.1016/j.bbrc.2005.11.021 16307728

[B365] Lorenz A. Deutschmann M. Ahlfeld J. Prix C. Koch A. Smits R.. (2011). Severe alterations of cerebellar cortical development after constitutive activation of Wnt signaling in granule neuron precursors. Mol. Cell. Biol. 31, 3326–3338. 10.1128/MCB.05718-11 21690300 PMC3147790

[B185] Lossi L. (2004). Occurrence of two different mechanisms of apoptosis in cerebellar granule cells in relation to the specificity of poly-ADP-ribose polymerase-1 (PARP) activation. Vet. Res. Commun. 28(Suppl. 1), 197–200. 10.1023/b:verc.0000045405.66323.4d 15372956

[B186] Lossi L. Coli A. Giannessi E. Stornelli M. R. Marroni P. (2002a). Cell proliferation and apoptosis during histogenesis of the guinea pig and rabbit cerebellar cortex. Ital. J. Anat. Embryol. 107, 117–125. 12113526

[B187] Lossi L. Merighi A. (2003). In vivo cellular and molecular mechanisms of neuronal apoptosis in the mammalian CNS. Prog. Neurobiol. 69, 287–312. 10.1016/s0301-0082(03)00051-0 12787572

[B188] Lossi L. Mioletti S. Merighi A. (2002b). Synapse-independent and synapse-dependent apoptosis of cerebellar granule cells in postnatal rabbits occur at two subsequent but partly overlapping developmental stages. Neuroscience 112, 509–523. 10.1016/s0306-4522(02)00112-4 12074894

[B189] Lossi L. Zagzag D. Greco M. A. Merighi A. (1998). Apoptosis of undifferentiated progenitors and granule cell precursors in the postnatal human cerebellar cortex correlates with expression of BCL-2, ICE and CPP32 proteins. J. Comp. Neurol. 399, 359–372. 10.1002/(sici)1096-9861(19980928)399:3<359::aid-cne5>3.0.co;2-# 9733083

[B190] Louvi A. Alexandre P. Metin C. Wurst W. Wassef M. (2003). The isthmic neuroepithelium is essential for cerebellar midline fusion. Development 130, 5319–5330. 10.1242/dev.00736 14507778

[B191] Lu S. Lu L. Y. Liu M. F. Yuan Q. J. Sham M. H. Guan X. Y.. (2012). Cerebellar defects in Pdss2 conditional knockout mice during embryonic development and in adulthood. Neurobiol. Dis. 45, 219–233. 10.1016/j.nbd.2011.08.006 21871565

[B192] Lumsden A. (2004). Segmentation and compartition in the early avian hindbrain. Mech. Dev. 121, 1081–1088. 10.1016/j.mod.2004.04.018 15296973

[B193] Luo S. Mao C. Lee B. Lee A. S. (2006). GRP78/BiP is required for cell proliferation and protecting the inner cell mass from apoptosis during early mouse embryonic development. Mol. Cell. Biol. 26, 5688–5697. 10.1128/MCB.00779-06 16847323 PMC1592753

[B194] Lutolf S. Radtke F. Aguet M. Suter U. Taylor V. (2002). Notch1 is required for neuronal and glial differentiation in the cerebellm. Development 129, 373–385. 11807030 10.1242/dev.129.2.373

[B195] Ma Q. Sommer L. Cserjesi P. Anderson D. J. (1997). Mash1 and neurogenin1 expression patterns define complementary domains of neuroepithelium in the developing CNS and are correlated with regions expressing notch ligands. J. Neurosci. 17, 3644–3652. 9133387 10.1523/JNEUROSCI.17-10-03644.1997 PMC6573688

[B196] Machold R. Fishell G. (2005). Math1 is expressed in temporally discrete pools of cerebellar rhombic-lip neural progenitors. Neuron 48, 17–24. 10.1016/j.neuron.2005.08.028 16202705

[B197] Mannan A. U. Roussa E. Kraus C. Rickmann M. Maenner J. Nayernia K.. (2004). Mutation in the gene encoding lysosomal acid phosphatase (Acp2) causes cerebellum and skin malformation in mouse. Neurogenetics 5, 229–238. 10.1007/s10048-004-0197-9 15503243

[B198] Manoranjan B. Venugopal C. Mcfarlane N. Doble B. W. Dunn S. E. Scheinemann K.. (2013). Medulloblastoma stem cells: modeling tumor heterogeneity. Cancer Lett. 338, 23–31. 10.1016/j.canlet.2012.07.010 22796365

[B199] Manto M. Gruol D. Schmahmann J. Koibuchi N. Rossi F. (2013). Handbook of the Cerebellum and Cerebellar Disorders. New York: Springer.

[B200] Marani E. Voogd J. (1979). The morphology of the mouse cerebellum. Acta Morphol. Neerl. Scand. 17, 33–52. 452954

[B201] Maricich S. M. Herrup K. (1999). Pax-2 expression defines a subset of GABAergic interneurons and their precursors in the developing murine cerebellum. J. Neurobiol. 41, 281–294. 10.1002/(sici)1097-4695(19991105)41:2<281::aid-neu10>3.0.co;2-5 10512984

[B202] Marín-Teva J. L. Dusart I. Colin C. Gervais A. van Rooijen N. Mallat M. (2004). Microglia promote the death of developing Purkinje cells. Neuron 41, 535–547. 10.1016/s0896-6273(04)00069-8 14980203

[B203] Marmolino D. Manto M. (2010). Past, present and future therapeutics for cerebellar ataxias. Curr. Neuropharmacol. 8, 41–61. 10.2174/157015910790909476 20808545 PMC2866461

[B204] Martínez S. (2001). The isthmic organizer and brain regionalization. Int. J. Dev. Biol. 45, 367–371. 11291867

[B205] Martinez S. Andreu A. Mecklenburg N. Echevarria D. (2013). Cellular and molecular basis of cerebellar development. Front. Neuroanat. 7:18. 10.3389/fnana.2013.00018 23805080 PMC3693072

[B206] Marzban H. Chung S. H. Pezhouh M. K. Feirabend H. Watanabe M. Voogd J.. (2010). Antigenic compartmentation of the cerebellar cortex in the chicken (Gallus domesticus). J. Comp. Neurol. 518, 2221–2239. 10.1002/cne.22328 20437525

[B207] Marzban H. Hawkes R. (2011). On the architecture of the posterior zone of the cerebellum. Cerebellum 10, 422–434. 10.1007/s12311-010-0208-3 20838950

[B208] Marzban H. Hoy N. Aavani T. Sarko D. K. Catania K. C. Hawkes R. (2011). Compartmentation of the cerebellar cortex in the naked mole-rat (Heterocephalus glaber). Cerebellum 10, 435–448. 10.1007/s12311-011-0251-8 21298580

[B209] Marzban H. Hoy N. Buchok M. Catania K. C. Hawkes R. (2014). Compartmentation of the cerebellar cortex: adaptation to lifestyle in the star-nosed mole Condylura cristata. Cerebellum [Epub ahead of print]. 10.1007/s12311-014-0618-8 25337886

[B210] Marzban H. Hoy N. Marotte L. R. Hawkes R. (2012). Antigenic compartmentation of the cerebellar cortex in an Australian marsupial, the tammar wallaby Macropus eugenii. Brain Behav. Evol. 80, 196–209. 10.1159/000340069 22907194

[B211] Marzban H. Kim C. T. Doorn D. Chung S. H. Hawkes R. (2008). A novel transverse expression domain in the mouse cerebellum revealed by a neurofilament-associated antigen. Neuroscience 153, 1190–1201. 10.1016/j.neuroscience.2008.02.036 18455884

[B212] Marzban H. Sillitoe R. V. Hoy M. Chung S. H. Rafuse V. F. Hawkes R. (2004). Abnormal HNK-1 expression in the cerebellum of an N-CAM null mouse. J. Neurocytol. 33, 117–130. 10.1023/b:neur.0000029652.96456.0d 15173636

[B213] Marzban H. Zahedi S. Sanchez M. Hawkes R. (2003). Antigenic compartmentation of the cerebellar cortex in the syrian hamster Mesocricetus auratus. Brain Res. 974, 176–183. 10.1016/s0006-8993(03)02576-9 12742635

[B214] Massey A. C. Zhang C. Cuervo A. M. (2006). Chaperone-mediated autophagy in aging and disease. Curr. Top. Dev. Biol. 73, 205–235. 10.1016/s0070-2153(05)73007-6 16782460

[B215] McMahon A. P. Joyner A. L. Bradley A. McMahon J. A. (1992). The midbrain-hindbrain phenotype of Wnt-1-/Wnt-1- mice results from stepwise deletion of engrailed-expressing cells by 9.5 days postcoitum. Cell 69, 581–595. 10.1016/0092-8674(92)90222-x 1534034

[B216] McNamee J. P. Bellier P. V. McLean J. R. Marro L. Gajda G. B. Thansandote A. (2002). DNA damage and apoptosis in the immature mouse cerebellum after acute exposure to a 1 mT, 60 Hz magnetic field. Mutat. Res. 513, 121–133. 10.1016/s1383-5718(01)00302-3 11719097

[B217] Meehan R. R. Lewis J. D. Bird A. P. (1992). Characterization of MeCP2, a vertebrate DNA binding protein with affinity for methylated DNA. Nucleic Acids Res. 20, 5085–5092. 10.1093/nar/20.19.5085 1408825 PMC334288

[B218] Miale I. L. Sidman R. L. (1961). An autoradiographic analysis of histogenesis in the mouse cerebellum. Exp. Neurol. 4, 277–296. 10.1016/0014-4886(61)90055-3 14473282

[B219] Millen K. J. Gleeson J. G. (2008). Cerebellar development and disease. Curr. Opin. Neurobiol. 18, 12–19. 10.1016/j.conb.2008.05.010 18513948 PMC2474776

[B220] Millen K. J. Hui C. C. Joyner A. L. (1995). A role for En-2 and other murine homologues of Drosophila segment polarity genes in regulating positional information in the developing cerebellum. Development 121, 3935–3945. 8575294 10.1242/dev.121.12.3935

[B221] Millen K. J. Steshina E. Y. Iskusnykh I. Y. Chizhikov V. V. (2014). Transformation of the cerebellum into more ventral brainstem fates causes cerebellar agenesis in the absence of Ptf1a function. Proc. Natl. Acad. Sci. U S A 111, E1777–1786. 10.1073/pnas.1315024111 24733890 PMC4035921

[B222] Millet S. Bloch-Gallego E. Simeone A. Alvarado-Mallart R. M. (1996). The caudal limit of Otx2 gene expression as a marker of the midbrain/hindbrain boundary: a study using in situ hybridisation and chick/quail homotopic grafts. Development 122, 3785–3797. 9012500 10.1242/dev.122.12.3785

[B223] Millonig J. H. Millen K. J. Hatten M. E. (2000). The mouse Dreher gene Lmx1a controls formation of the roof plate in the vertebrate CNS. Nature 403, 764–769. 10.1038/35001573 10693804

[B224] Milosevic A. Zecevic N. (1998). Developmental changes in human cerebellum: expression of intracellular calcium receptors, calcium-binding proteins and phosphorylated and nonphosphorylated neurofilament protein. J. Comp. Neurol. 396, 442–460. 10.1002/(sici)1096-9861(19980713)396:4<442::aid-cne3>3.3.co;2-# 9651004

[B225] Miyata T. Ono Y. Okamoto M. Masaoka M. Sakakibara A. Kawaguchi A.. (2010). Migration, early axonogenesis and Reelin-dependent layer-forming behavior of early/posterior-born Purkinje cells in the developing mouse lateral cerebellum. Neural Dev. 5:23. 10.1186/1749-8104-5-23 20809939 PMC2942860

[B226] Mnatzakanian G. N. Lohi H. Munteanu I. Alfred S. E. Yamada T. MacLeod P. J.. (2004). A previously unidentified MECP2 open reading frame defines a new protein isoform relevant to Rett syndrome. Nat. Genet. 36, 339–341. 10.1038/ng1327 15034579

[B227] Mochida G. H. Ganesh V. S. de Michelena M. I. Dias H. Atabay K. D. Kathrein K. L.. (2012). CHMP1A encodes an essential regulator of BMI1-INK4A in cerebellar development. Nat. Genet. 44, 1260–1264. 10.1038/ng.2425 23023333 PMC3567443

[B228] Montgomery J. C. Bodznick D. Yopak K. E. (2012). The cerebellum and cerebellum-like structures of cartilaginous fishes. Brain Behav. Evol. 80, 152–165. 10.1159/000339868 22986830

[B229] Mordel J. Karnas D. Pévet P. Isope P. Challet E. Meissl H. (2013). The output signal of Purkinje cells of the cerebellum and circadian rhythmicity. PLoS One 8:e58457. 10.1371/journal.pone.0058457 23505510 PMC3591352

[B230] Moreno S. Imbroglini V. Ferraro E. Bernardi C. Romagnoli A. Berrebi A. S.. (2006). Apoptosome impairment during development results in activation of an autophagy program in cerebral cortex. Apoptosis 11, 1595–1602. 10.1007/s10495-006-9081-4 16820961

[B231] Mugnaini E. Floris A. (1994). The unipolar brush cell: a neglected neuron of the mammalian cerebellar cortex. J. Comp. Neurol. 339, 174–180. 10.1002/cne.903390203 8300904

[B232] Mugnaini E. Sekerková G. Martina M. (2011). The unipolar brush cell: a remarkable neuron finally receiving deserved attention. Brain Res. Rev. 66, 220–245. 10.1016/j.brainresrev.2010.10.001 20937306 PMC3030675

[B233] Müller F. O’Rahilly R. (1988a). The development of the human brain from a closed neural tube at stage 13. Anat. Embryol. (Berl) 177, 203–224. 10.1007/bf00321132 3354839

[B234] Müller F. O’Rahilly R. (1988b). The development of the human brain, including the longitudinal zoning in the diencephalon at stage 15. Anat. Embryol. (Berl) 179, 55–71. 10.1007/bf00305100 3213956

[B235] Müller F. O’Rahilly R. (1989). The human brain at stage 16, including the initial evagination of the neurohypophysis. Anat. Embryol. (Berl) 179, 551–569. 10.1007/bf00315698 2751117

[B236] Müller F. O’Rahilly R. (1990a). The human brain at stages 18–20, including the choroid plexuses and the amygdaloid and septal nuclei. Anat. Embryol. (Berl) 182, 285–306. 10.1007/bf00185521 2268071

[B237] Müller F. O’Rahilly R. (1990b). The human brain at stages 21–23, with particular reference to the cerebral cortical plate and to the development of the cerebellum. Anat. Embryol. (Berl) 182, 375–400. 10.1007/bf02433497 2252222

[B238] Murakami J. W. Courchesne E. Haas R. H. Press G. A. Yeung-Courchesne R. (1992). Cerebellar and cerebral abnormalities in Rett syndrome: a quantitative MR analysis. AJR Am. J. Roentgenol. 159, 177–183. 10.2214/ajr.159.1.1609693 1609693

[B239] Nat R. Voiculescu B. Stanciu C. Vidulescu C. Cergan R. Badiu C.. (2001). Apoptosis in human embryo development: 2. Cerebellum. J. Cell. Mol. Med. 5, 179–187. 10.1111/j.1582-4934.2001.tb00151.x 12067506 PMC6738124

[B240] Ni M. Lee A. S. (2007). ER chaperones in mammalian development and human diseases. FEBS Lett. 581, 3641–3651. 10.1016/j.febslet.2007.04.045 17481612 PMC2040386

[B241] Nolte C. Rastegar M. Amores A. Bouchard M. Grote D. Maas R.. (2006). Stereospecificity and PAX6 function direct Hoxd4 neural enhancer activity along the antero-posterior axis. Dev. Biol. 299, 582–593. 10.1016/j.ydbio.2006.08.061 17010333

[B242] Northcutt R. G. (2002). Understanding vertebrate brain evolution. Integr. Comp. Biol. 42, 743–756. 10.1093/icb/42.4.743 21708771

[B243] Nowakowska-Kotas M. Kedzia A. Dudek K. (2014). Development of external surfaces of human cerebellar lobes in the fetal period. Cerebellum 13, 541–548. 10.1007/s12311-014-0566-3 24831768 PMC4155164

[B244] Okano-Uchida T. Himi T. Komiya Y. Ishizaki Y. (2004). Cerebellar granule cell precursors can differentiate into astroglial cells. Proc. Natl. Acad. Sci. U S A 101, 1211–1216. 10.1073/pnas.0307972100 14745007 PMC337032

[B245] Oldfors A. Sourander P. Armstrong D. L. Percy A. K. Witt-Engerstrom I. Hagberg B. A. (1990). Rett syndrome: cerebellar pathology. Pediatr. Neurol. 6, 310–314. 10.1016/0887-8994(90)90022-s 2242172

[B246] Olsen L. Klausen M. Helboe L. Nielsen F. C. Werge T. (2009). MicroRNAs show mutually exclusive expression patterns in the brain of adult male rats. PLoS One 4:e7225. 10.1371/journal.pone.0007225 19806225 PMC2752988

[B247] Olson C. O. Zachariah R. M. Ezeonwuka C. D. Liyanage V. R. Rastegar M. (2014). Brain region-specific expression of MeCP2 isoforms correlates with DNA methylation within Mecp2 regulatory elements. PLoS One 9:e90645. 10.1371/journal.pone.0090645 24594659 PMC3940938

[B248] Olynik B. M. Rastegar M. (2012). The genetic and epigenetic journey of embryonic stem cells into mature neural cells. Front. Genet. 3:81. 10.3389/fgene.2012.00081 22629283 PMC3355330

[B249] Oomman S. Finckbone V. Dertien J. Attridge J. Henne W. Medina M.. (2004). Active caspase-3 expression during postnatal development of rat cerebellum is not systematically or consistently associated with apoptosis. J. Comp. Neurol. 476, 154–173. 10.1002/cne.20223 15248196

[B250] Oomman S. Strahlendorf H. Finckbone V. Strahlendorf J. (2005). Non-lethal active caspase-3 expression in Bergmann glia of postnatal rat cerebellum. Brain Res. Dev. Brain Res. 160, 130–145. 10.1016/j.devbrainres.2005.07.010 16226814

[B251] Oppenheim R. W. (1991). Cell death during development of the nervous system. Annu. Rev. Neurosci. 14, 453–501. 10.1146/annurev.neuro.14.1.453 2031577

[B252] Palmen S. J. Van Engeland H. Hof P. R. Schmitz C. (2004). Neuropathological findings in autism. Brain 127, 2572–2583. 10.1093/brain/awh287 15329353

[B253] Partanen J. (2007). FGF signalling pathways in development of the midbrain and anterior hindbrain. J. Neurochem. 101, 1185–1193. 10.1111/j.1471-4159.2007.04463.x 17326764

[B254] Patil N. Cox D. R. Bhat D. Faham M. Myers R. M. Peterson A. S. (1995). A potassium channel mutation in weaver mice implicates membrane excitability in granule cell differentiation. Nat. Genet. 11, 126–129. 10.1038/ng1095-126 7550338

[B255] Pidsley R. Dempster E. Troakes C. Al-Sarraj S. Mill J. (2012a). Epigenetic and genetic variation at the IGF2/H19 imprinting control region on 11p15.5 is associated with cerebellum weight. Epigenetics 7, 155–163. 10.4161/epi.7.2.18910 22395465 PMC3335909

[B256] Pidsley R. Fernandes C. Viana J. Paya-Cano J. L. Liu L. Smith R. G.. (2012b). DNA methylation at the Igf2/H19 imprinting control region is associated with cerebellum mass in outbred mice. Mol. Brain 5:42. 10.1186/1756-6606-5-42 23216893 PMC3541153

[B366] Plummer J. T. Evgrafov O. V. Bergman M. Y. Friez M. Haiman C. A. Levitt P.. (2013). Transcriptional regulation of the MET receptor tyrosine kinase gene by MeCP2 and sex-specific expression in autism and Rett syndrome. Transl. Psychiatry 3:e316. 10.1038/tp.2013.91 24150225 PMC3818007

[B257] Provenzano G. Pangrazzi L. Poli A. Sgado P. Berardi N. Bozzi Y. (2014). Reduced phosphorylation of synapsin I in the hippocampus of Engrailed-2 knockout mice, a model for autism spectrum disorders. Neuroscience 286C, 122–130. 10.1016/j.neuroscience.2014.11.041 25463523

[B258] Quartu M. Serra M. P. Manca A. Follesa P. Ambu R. Del Fiacco M. (2003a). High affinity neurotrophin receptors in the human pre-term newborn, infant and adult cerebellum. Int. J. Dev. Neurosci. 21, 309–320. 10.1016/s0736-5748(03)00086-8 12927579

[B259] Quartu M. Serra M. P. Manca A. Follesa P. Lai M. L. Del Fiacco M. (2003b). Neurotrophin-like immunoreactivity in the human pre-term newborn, infant and adult cerebellum. Int. J. Dev. Neurosci. 21, 23–33. 10.1016/s0736-5748(02)00110-7 12565693

[B260] Rabié A. Favre C. Clavel M. C. Legrand J. (1977). Effects of thyroid dysfunction on the development of the rat cerebellum, with special reference to cell death within the internal granular layer. Brain Res. 120, 521–531. 10.1016/0006-8993(77)90405-x 832138

[B261] Raff M. C. Barres B. A. Burne J. F. Coles H. S. Ishizaki Y. Jacobson M. D. (1993). Programmed cell death and the control of cell survival: lessons from the nervous system. Science 262, 695–700. 10.1126/science.8235590 8235590

[B262] Rakic P. (1972). Extrinsic cytological determinants of basket and stellate cell dendritic pattern in the cerebellar molecular layer. J. Comp. Neurol. 146, 335–354. 10.1002/cne.901460304 4628749

[B263] Rakic P. Sidman R. L. (1970). Histogenesis of cortical layers in human cerebellum, particularly the lamina dissecans. J. Comp. Neurol. 139, 473–500. 10.1002/cne.901390407 4195699

[B264] Rami A. (2009). Review: autophagy in neurodegeneration: firefighter and/or incendiarist? Neuropathol. Appl. Neurobiol. 35, 449–461. 10.1111/j.1365-2990.2009.01034.x 19555462

[B265] Rastegar M. Hotta A. Pasceri P. Makarem M. Cheung A. Y. Elliott S.. (2009). MECP2 isoform-specific vectors with regulated expression for Rett syndrome gene therapy. PLoS One 4:e6810. 10.1371/journal.pone.0006810 19710912 PMC2728539

[B266] Rastegar M. Kobrossy L. Kovacs E. N. Rambaldi I. Featherstone M. (2004). Sequential histone modifications at Hoxd4 regulatory regions distinguish anterior from posterior embryonic compartments. Mol. Cell. Biol. 24, 8090–8103. 10.1128/mcb.24.18.8090-8103.2004 15340071 PMC515066

[B267] Rastegar M. Lemaigre F. P. Rousseau G. G. (2000a). Control of gene expression by growth hormone in liver: key role of a network of transcription factors. Mol. Cell. Endocrinol. 164, 1–4. 10.1016/s0303-7207(00)00263-x 11026552

[B268] Rastegar M. Rousseau G. G. Lemaigre F. P. (2000b). CCAAT/enhancer-binding protein-alpha is a component of the growth hormone-regulated network of liver transcription factors. Endocrinology 141, 1686–1692. 10.1210/en.141.5.1686 10803577

[B269] Reeber S. L. Otis T. S. Sillitoe R. V. (2013). New roles for the cerebellum in health and disease. Front. Syst. Neurosci. 7:83. 10.3389/fnsys.2013.00083 24294192 PMC3827539

[B270] Rhinn M. Brand M. (2001). The midbrain–hindbrain boundary organizer. Curr. Opin. Neurobiol. 11, 34–42. 10.1016/S0959-4388(00)00171-9 11179870

[B271] Robey E. (1997). Notch in vertebrates. Curr. Opin. Genet. Dev. 7, 551–557. 10.1016/s0959-437x(97)80085-8 9309189

[B272] Rogers T. D. Mckimm E. Dickson P. E. Goldowitz D. Blaha C. D. Mittleman G. (2013). Is autism a disease of the cerebellum? An integration of clinical and pre-clinical research. Front. Syst. Neurosci. 7:15. 10.3389/fnsys.2013.00015 23717269 PMC3650713

[B273] Ronan M. Northcutt R. (1998). “The central nervous system of hagfishes,” in The Biology of Hagfishes ed Farrell A. P. (London: Chapman and Hall), 454–478.

[B274] Ross M. E. Fletcher C. Mason C. A. Hatten M. E. Heintz N. (1990). Meander tail reveals a discrete developmental unit in the mouse cerebellum. Proc. Natl. Acad. Sci. U S A 87, 4189–4192. 10.1073/pnas.87.11.4189 2349228 PMC54073

[B275] Ruigrok T. J. (1997). Cerebellar nuclei: the olivary connection. Prog. Brain Res. 114, 167–192. 10.1016/s0079-6123(08)63364-6 9193144

[B276] Rust M. B. Kullmann J. A. Witke W. (2012). Role of the actin-binding protein profilin1 in radial migration and glial cell adhesion of granule neurons in the cerebellum. Cell Adh. Migr. 6, 13–17. 10.4161/cam.19845 22647936 PMC3364132

[B277] Sajan S. A. Waimey K. E. Millen K. J. (2010). Novel approaches to studying the genetic basis of cerebellar development. Cerebellum 9, 272–283. 10.1007/s12311-010-0169-6 20387026 PMC2921561

[B278] Sandhu S. Wu X. Nabi Z. Rastegar M. Kung S. Mai S.. (2012). Loss of HLTF function promotes intestinal carcinogenesis. Mol. Cancer 11:18. 10.1186/1476-4598-11-18 22452792 PMC3337324

[B279] Sawada K. Fukui Y. Hawkes R. (2008). Spatial distribution of corticotropin-releasing factor immunopositive climbing fibers in the mouse cerebellum: analysis by whole mount immunohistochemistry. Brain Res. 1222, 106–117. 10.1016/j.brainres.2008.05.029 18572150

[B280] Schaefer A. O’carroll D. Tan C. L. Hillman D. Sugimori M. Llinas R.. (2007). Cerebellar neurodegeneration in the absence of microRNAs. J. Exp. Med. 204, 1553–1558. 10.1084/jem.20070823 17606634 PMC2118654

[B281] Schmahmann J. D. (1997). Rediscovery of an early concept. Int. Rev. Neurobiol. 41, 3–27. 10.1016/s0074-7742(08)60345-1 9378594

[B282] Schmahmann J. D. Caplan D. (2006). Cognition, emotion and the cerebellum. Brain 129, 290–292. 10.1093/brain/awh729 16434422

[B283] Schuurmans C. Armant O. Nieto M. Stenman J. M. Britz O. Klenin N.. (2004). Sequential phases of cortical specification involve Neurogenin-dependent and -independent pathways. EMBO J. 23, 2892–2902. 10.1038/sj.emboj.7600278 15229646 PMC514942

[B284] Schweighofer N. Doya K. Kuroda S. (2004). Cerebellar aminergic neuromodulation: towards a functional understanding. Brain Res. Brain Res. Rev. 44, 103–116. 10.1016/j.brainresrev.2003.10.004 15003388

[B285] Sekerková G. Ilijic E. Mugnaini E. (2004). Bromodeoxyuridine administered during neurogenesis of the projection neurons causes cerebellar defects in rat. J. Comp. Neurol. 470, 221–239. 10.1002/cne.11016 14755513

[B286] Senderek J. Krieger M. Stendel C. Bergmann C. Moser M. Breitbach-Faller N.. (2005). Mutations in SIL1 cause Marinesco-Sjogren syndrome, a cerebellar ataxia with cataract and myopathy. Nat. Genet. 37, 1312–1314. 10.1038/ng1678 16282977

[B287] Sgaier S. K. Millet S. Villanueva M. P. Berenshteyn F. Song C. Joyner A. L. (2005). Morphogenetic and cellular movements that shape the mouse cerebellum; insights from genetic fate mapping. Neuron 45, 27–40. 10.1016/j.neuron.2004.12.021 15629700

[B288] Shamim H. Mason I. (1998). Expression of Gbx-2 during early development of the chick embryo. Mech. Dev. 76, 157–159. 10.1016/s0925-4773(98)00102-6 9767156

[B289] Shevelkin A. V. Ihenatu C. Pletnikov M. V. (2014). Pre-clinical models of neurodevelopmental disorders: focus on the cerebellum. Rev. Neurosci. 25, 177–194. 10.1515/revneuro-2013-0049 24523305 PMC4052755

[B290] Silbereis J. Heintz T. Taylor M. M. Ganat Y. Ment L. R. Bordey A.. (2010). Astroglial cells in the external granular layer are precursors of cerebellar granule neurons in neonates. Mol. Cell Neurosci. 44, 362–373. 10.1016/j.mcn.2010.05.001 20470892 PMC2900521

[B291] Sillitoe R. V. Joyner A. L. (2007). Morphology, molecular codes and circuitry produce the three-dimensional complexity of the cerebellum. Annu. Rev. Cell Dev. Biol. 23, 549–577. 10.1146/annurev.cellbio.23.090506.123237 17506688

[B292] Simonati A. Rosso T. Rizzuto N. (1997). DNA fragmentation in normal development of the human central nervous system: a morphological study during corticogenesis. Neuropathol. Appl. Neurobiol. 23, 203–211. 10.1111/j.1365-2990.1997.tb01203.x 9223129

[B293] Simonati A. Tosati C. Rosso T. Piazzola E. Rizzuto N. (1999). Cell proliferation and death: morphological evidence during corticogenesis in the developing human brain. Microsc. Res. Tech. 45, 341–352. 10.1002/(sici)1097-0029(19990615)45:6<341::aid-jemt2>3.0.co;2-u 10402262

[B294] Sinha R. A. Khare P. Rai A. Maurya S. K. Pathak A. Mohan V.. (2009). Anti-apoptotic role of omega-3-fatty acids in developing brain: perinatal hypothyroid rat cerebellum as apoptotic model. Int. J. Dev. Neurosci. 27, 377–383. 10.1016/j.ijdevneu.2009.02.003 19460632

[B295] Smeyne R. J. Goldowitz D. (1989). Development and death of external granular layer cells in the weaver mouse cerebellum: a quantitative study. J. Neurosci. 9, 1608–1620. 2723742 10.1523/JNEUROSCI.09-05-01608.1989 PMC6569844

[B296] Sohma O. Mizuguchi M. Takashima S. Yamada M. Ikeda K. Ohta S. (1996). High expression of Bcl-x protein in the developing human cerebellar cortex. J. Neurosci. Res 43, 175–182. 10.1002/(sici)1097-4547(19960115)43:2<175::aid-jnr5>3.0.co;2-d 8820965

[B297] Sotelo C. Wassef M. (1991). Cerebellar development: afferent organization and Purkinje cell heterogeneity. Philos. Trans. R. Soc. Lond. B Biol. Sci. 331, 307–313. 10.1098/rstb.1991.0022 1677476

[B298] Steuber V. Jaeger D. (2013). Modeling the generation of output by the cerebellar nuclei. Neural Netw. 47, 112–119. 10.1016/j.neunet.2012.11.006 23200193 PMC3596440

[B299] Stoykova A. Gruss P. (1994). Roles of Pax-genes in developing and adult brain as suggested by expression patterns. J. Neurosci. 14, 1395–1412. 8126546 10.1523/JNEUROSCI.14-03-01395.1994 PMC6577564

[B300] Striedter G. F. (2005). Principles of Brain Evolution. Sunderland, MA: Sinauer Associates.

[B301] Sugihara I. Quy P. N. (2007). Identification of aldolase C compartments in the mouse cerebellar cortex by olivocerebellar labeling. J. Comp. Neurol. 500, 1076–1092. 10.1002/cne.21219 17183552

[B302] Sun E. Shi Y. (2014). MicroRNAs: small molecules with big roles in neurodevelopment and diseases. Exp. Neurol. [Epub ahead of print]. 10.1016/j.expneurol.2014.08.005. 25128264

[B303] Szulwach K. E. Li X. Li Y. Song C. X. Wu H. Dai Q.. (2011). 5-hmC-mediated epigenetic dynamics during postnatal neurodevelopment and aging. Nat. Neurosci. 14, 1607–1616. 10.1038/nn.2959 22037496 PMC3292193

[B304] Tamura T. Said S. Lu W. Neufeld D. (2000). Specificity of TUNEL method depends on duration of fixation. Biotech. Histochem. 75, 197–200. 10.3109/10520290009066501 10999571

[B305] Tanaka M. Marunouchi T. (1998). Immunohistochemical analysis of developmental stage of external granular layer neurons which undergo apoptosis in postnatal rat cerebellum. Neurosci. Lett. 242, 85–88. 10.1016/s0304-3940(98)00032-9 9533400

[B306] Tao J. Wu H. Lin Q. Wei W. Lu X. H. Cantle J. P.. (2011). Deletion of astroglial Dicer causes non-cell-autonomous neuronal dysfunction and degeneration. J. Neurosci. 31, 8306–8319. 10.1523/jneurosci.0567-11.2011 21632951 PMC3500097

[B307] Taranukhin A. G. Taranukhina E. Y. Saransaari P. Podkletnova I. M. Pelto-Huikko M. Oja S. S. (2010). Neuroprotection by taurine in ethanol-induced apoptosis in the developing cerebellum. J. Biomed. Sci. 17(Suppl. 1):S12. 10.1186/1423-0127-17-s1-s12 20804586 PMC2994388

[B308] Tomoda T. Bhatt R. S. Kuroyanagi H. Shirasawa T. Hatten M. E. (1999). A mouse serine/threonine kinase homologous to *C. elegans* UNC51 functions in parallel fiber formation of cerebellar granule neurons. Neuron 24, 833–846. 10.1016/s0896-6273(00)81031-4 10624947

[B309] Topka S. Glassmann A. Weisheit G. Schuller U. Schilling K. (2014). The transcription factor Cux1 in cerebellar granule cell development and medulloblastoma pathogenesis. Cerebellum 13, 698–712. 10.1007/s12311-014-0588-x 25096634

[B310] Tsekhmistrenko T. A. (1999). Quantitative changes in human cerebellar pyriform neurons from birth to the age of 20 years. Neurosci. Behav. Physiol. 29, 405–409. 10.1007/bf02461076 10582222

[B311] Tsekhmistrenko T. A. (2001). Modular organization of the granular layer of the human cerebellar cortex during post-natal ontogenesis. Neurosci. Behav. Physiol. 31, 105–109. 10.1023/A:1026694818586 11265807

[B313] Uusisaari M. Y. Knöpfel T. (2012). Diversity of neuronal elements and circuitry in the cerebellar nuclei. Cerebellum 11, 420–421. 10.1007/s12311-011-0350-6 22278661

[B312] Uusisaari M. Obata K. Knopfel T. (2007). Morphological and electrophysiological properties of GABAergic and non-GABAergic cells in the deep cerebellar nuclei. J. Neurophysiol. 97, 901–911. 10.1152/jn.00974.2006 17093116

[B314] Valle M. S. Garifoli A. Maci T. Perciavalle V. (2001). Reticulocerebellar projections to the anterior and posterior lobes of the rat cerebellum. Neurosci. Lett. 314, 41–44. 10.1016/s0304-3940(01)02278-9 11698142

[B315] Vidovic M. Chen M. M. Lu Q. Y. Kalloniatis K. F. Martin B. M. Tan A. H.. (2008). Deficiency in endothelin receptor B reduces proliferation of neuronal progenitors and increases apoptosis in postnatal rat cerebellum. Cell Mol. Neurobiol. 28, 1129–1138. 10.1007/s10571-008-9292-z 18683040 PMC11515047

[B316] Vieira C. Pombero A. Garcia-Lopez R. Gimeno L. Echevarria D. Martinez S. (2010). Molecular mechanisms controlling brain development: an overview of neuroepithelial secondary organizers. Int. J. Dev. Biol. 54, 7–20. 10.1387/ijdb.092853cv 19876817

[B317] Voogd J. (2011). Cerebellar zones: a personal history. Cerebellum 10, 334–350. 10.1007/s12311-010-0221-6 20967577 PMC3169774

[B318] Voogd J. Gerrits N. M. Ruigrok T. J. (1996). Organization of the vestibulocerebellum. Ann. N Y Acad. Sci. 781, 553–579. 10.1111/j.1749-6632.1996.tb15728.x 8694444

[B319] Voogd J. Glickstein M. (1998). The anatomy of the cerebellum. Trends Cogn. Sci. 2, 307–313. 10.1016/S1364-6613(98)01210-8 21227226

[B320] Voogd J. Pardoe J. Ruigrok T. J. Apps R. (2003). The distribution of climbing and mossy fiber collateral branches from the copula pyramidis and the paramedian lobule: congruence of climbing fiber cortical zones and the pattern of zebrin banding within the rat cerebellum. J. Neurosci. 23, 4645–4656. 12805304 10.1523/JNEUROSCI.23-11-04645.2003 PMC6740790

[B322] Wang T. Pan Q. Lin L. Szulwach K. E. Song C. X. He C.. (2012a). Genome-wide DNA hydroxymethylation changes are associated with neurodevelopmental genes in the developing human cerebellum. Hum. Mol. Genet. 21, 5500–5510. 10.1093/hmg/dds394 23042784 PMC3516134

[B323] Wang V. Y. Rose M. F. Zoghbi H. Y. (2005). Math1 expression redefines the rhombic lip derivatives and reveals novel lineages within the brainstem and cerebellum. Neuron 48, 31–43. 10.1016/j.neuron.2005.08.024 16202707

[B321] Wang J. Wechsler-Reya R. J. (2014). The role of stem cells and progenitors in the genesis of medulloblastoma. Exp. Neurol. 260, 69–73. 10.1016/j.expneurol.2012.11.014 23178582 PMC3718859

[B325] Wang Y. Zhong J. Xu H. Wei W. Dong J. Yu F.. (2012b). Perinatal iodine deficiency and hypothyroidism increase cell apoptosis and alter doublecortin and reelin protein expressions in rat cerebellum. Arch. Med. Res. 43, 255–264. 10.1016/j.arcmed.2012.05.002 22595232

[B324] Wang V. Y. Zoghbi H. Y. (2001). Genetic regulation of cerebellar development. Nat. Rev. Neurosci. 2, 484–491. 10.1038/35081558 11433373

[B326] Wassarman K. M. Lewandoski M. Campbell K. Joyner A. L. Rubenstein J. L. Martinez S.. (1997). Specification of the anterior hindbrain and establishment of a normal mid/hindbrain organizer is dependent on Gbx2 gene function. Development 124, 2923–2934. 9247335 10.1242/dev.124.15.2923

[B327] Webb S. J. Sparks B. F. Friedman S. D. Shaw D. W. Giedd J. Dawson G.. (2009). Cerebellar vermal volumes and behavioral correlates in children with autism spectrum disorder. Psychiatry Res. 172, 61–67. 10.1016/j.pscychresns.2008.06.001 19243924 PMC2676721

[B328] Wechsler-Reya R. J. Scott M. P. (1999). Control of neuronal precursor proliferation in the cerebellum by Sonic Hedgehog. Neuron 22, 103–114. 10.1016/s0896-6273(00)80682-0 10027293

[B329] Wegiel J. Flory M. Kuchna I. Nowicki K. Ma S. Imaki H.. (2014). Stereological study of the neuronal number and volume of 38 brain subdivisions of subjects diagnosed with autism reveals significant alterations restricted to the striatum, amygdala and cerebellum. Acta Neuropathol. Commun. 2:141. 10.1186/s40478-014-0141-7 25231243 PMC4177256

[B330] Weigle C. Northcutt R. G. (1999). The chemoarchitecture of the forebrain of lampreys: evolutionary implications by comparisons with gnathostomes. Eur. J. Morphol. 37, 122–125. 10.1076/ejom.37.2-3.0122 10342442

[B331] White J. J. Sillitoe R. V. (2013a). Development of the cerebellum: from gene expression patterns to circuit maps. Wiley Interdiscip. Rev. Dev. Biol. 2, 149–164. 10.1002/wdev.65 23799634

[B332] White J. J. Sillitoe R. V. (2013b). Postnatal development of cerebellar zones revealed by neurofilament heavy chain protein expression. Front. Neuroanat. 7:9. 10.3389/fnana.2013.00009 23675325 PMC3648691

[B333] Wingate R. (2005). Math-Map(ic)s. Neuron 48, 1–4. 10.1016/j.neuron.2005.09.012 16202701

[B334] Wingate R. J. (2001). The rhombic lip and early cerebellar development. Curr. Opin. Neurobiol. 11, 82–88. 10.1016/s0959-4388(00)00177-x 11179876

[B335] Wingate R. J. Hatten M. E. (1999). The role of the rhombic lip in avian cerebellum development. Development 126, 4395–4404. 10498676 10.1242/dev.126.20.4395

[B336] Wirth E. K. Bharathi B. S. Hatfield D. Conrad M. Brielmeier M. Schweizer U. (2014). Cerebellar hypoplasia in mice lacking selenoprotein biosynthesis in neurons. Biol. Trace Elem. Res. 158, 203–210. 10.1007/s12011-014-9920-z 24599700 PMC3984410

[B337] Wiser A. K. Andreasen N. C. O’leary D. S. Watkins G. L. Boles Ponto L. L. Hichwa R. D. (1998). Dysfunctional cortico-cerebellar circuits cause ‘cognitive dysmetria’ in schizophrenia. Neuroreport 9, 1895–1899. 10.1097/00001756-199806010-00042 9665622

[B338] Wood K. A. Dipasquale B. Youle R. J. (1993). In situ labeling of granule cells for apoptosis-associated DNA fragmentation reveals different mechanisms of cell loss in developing cerebellum. Neuron 11, 621–632. 10.1016/0896-6273(93)90074-2 8398151

[B339] Wood K. A. Youle R. J. (1995). The role of free radicals and p53 in neuron apoptosis in vivo. J. Neurosci. 15, 5851–5857. 7643225 10.1523/JNEUROSCI.15-08-05851.1995 PMC6577632

[B340] Wullner U. Loschmann P. A. Weller M. Klockgether T. (1995). Apoptotic cell death in the cerebellum of mutant weaver and lurcher mice. Neurosci. Lett. 200, 109–112. 10.1016/0304-3940(95)12090-q 8614556

[B341] Wurst W. Bally-Cuif L. (2001). Neural plate patterning: upstream and downstream of the isthmic organizer. Nat. Rev. Neurosci. 2, 99–108. 10.1038/35053516 11253000

[B342] Xin Y. Chanrion B. Liu M. M. Galfalvy H. Costa R. Ilievski B.. (2010). Genome-wide divergence of DNA methylation marks in cerebral and cerebellar cortices. PLoS One 5:e11357. 10.1371/journal.pone.0011357 20596539 PMC2893206

[B344] Xu Z. Q. Sun Y. Li H. Y. Lim Y. Zhong J. H. Zhou X. F. (2011). Endogenous proBDNF is a negative regulator of migration of cerebellar granule cells in neonatal mice. Eur. J. Neurosci. 33, 1376–1384. 10.1111/j.1460-9568.2011.07635.x 21366730

[B343] Xu H. Yang Y. Tang X. Zhao M. Liang F. Xu P.. (2013). Bergmann glia function in granule cell migration during cerebellum development. Mol. Neurobiol. 47, 833–844. 10.1007/s12035-013-8405-y 23329344

[B345] Yachnis A. T. Rorke L. B. Lee V. M. Trojanowski J. Q. (1993). Expression of neuronal and glial polypeptides during histogenesis of the human cerebellar cortex including observations on the dentate nucleus. J. Comp. Neurol. 334, 356–369. 10.1002/cne.903340303 7690783

[B346] Yamada K. Watanabe M. (2002). Cytodifferentiation of Bergmann glia and its relationship with Purkinje cells. Anat. Sci. Int. 77, 94–108. 10.1046/j.0022-7722.2002.00021.x 12418089

[B347] Yang Z. Klionsky D. J. (2010). Eaten alive: a history of macroautophagy. Nat. Cell Biol. 12, 814–822. 10.1038/ncb0910-814 20811353 PMC3616322

[B348] Yao B. Lin L. Street R. C. Zalewski Z. A. Galloway J. N. Wu H.. (2014). Genome-wide alteration of 5-hydroxymethylcytosine in a mouse model of fragile X-associated tremor/ataxia syndrome. Hum. Mol. Genet. 23, 1095–1107. 10.1093/hmg/ddt504 24108107 PMC3900112

[B349] Yasui D. H. Gonzales M. L. Aflatooni J. O. Crary F. K. Hu D. J. Gavino B. J.. (2014). Mice with an isoform-ablating Mecp2 exon 1 mutation recapitulate the neurologic deficits of Rett syndrome. Hum. Mol. Genet. 23, 2447–2458. 10.1093/hmg/ddt640 24352790 PMC3976336

[B350] Yeung J. Ha T. J. Swanson D. J. Choi K. Tong Y. Goldowitz D. (2014). Wls provides a new compartmental view of the rhombic lip in mouse cerebellar development. J. Neurosci. 34, 12527–12537. 10.1523/jneurosci.1330-14.2014 25209290 PMC4160781

[B351] Yokota N. Aruga J. Takai S. Yamada K. Hamazaki M. Iwase T.. (1996). Predominant expression of human zic in cerebellar granule cell lineage and medulloblastoma. Cancer Res. 56, 377–383. 8542595

[B352] Yopak K. E. (2012). Neuroecology of cartilaginous fishes: the functional implications of brain scaling. J. Fish Biol. 80, 1968–2023. 10.1111/j.1095-8649.2012.03254.x 22497414

[B353] Yopak K. E. Montgomery J. C. (2008). Brain organization and specialization in deep-sea chondrichthyans. Brain Behav. Evol. 71, 287–304. 10.1159/000127048 18431055

[B354] Yu T. Meiners L. C. Danielsen K. Wong M. T. Bowler T. Reinberg D.. (2013). Deregulated FGF and homeotic gene expression underlies cerebellar vermis hypoplasia in CHARGE syndrome. Elife 2:e01305. 10.7554/elife.01305 24368733 PMC3870572

[B355] Yuasa S. (1996). Bergmann glial development in the mouse cerebellum as revealed by tenascin expression. Anat. Embryol. (Berl) 194, 223–234. 10.1007/bf00187133 8849669

[B356] Zachariah R. M. Olson C. O. Ezeonwuka C. Rastegar M. (2012). Novel MeCP2 isoform-specific antibody reveals the endogenous MeCP2E1 expression in murine brain, primary neurons and astrocytes. PLoS One 7:e49763. 10.1371/journal.pone.0049763 23185431 PMC3501454

[B357] Zachariah R. M. Rastegar M. (2012). Linking epigenetics to human disease and Rett syndrome: the emerging novel and challenging concepts in MeCP2 research. Neural Plast. 2012:415825. 10.1155/2012/415825 22474603 PMC3306986

[B358] Zanni G. Barresi S. Travaglini L. Bernardini L. Rizza T. Digilio M. C.. (2011). FGF17, a gene involved in cerebellar development, is downregulated in a patient with Dandy-Walker malformation carrying a de novo 8p deletion. Neurogenetics 12, 241–245. 10.1007/s10048-011-0283-8 21484435

[B359] Zecevic N. Rakic P. (1976). Differentiation of Purkinje cells and their relationship to other components of developing cerebellar cortex in man. J. Comp. Neurol. 167, 27–47. 10.1002/cne.901670103 818132

[B360] Zhang L. Goldman J. E. (1996). Developmental fates and migratory pathways of dividing progenitors in the postnatal rat cerebellum. J. Comp. Neurol. 370, 536–550. 10.1002/(sici)1096-9861(19960708)370:4<536::aid-cne9>3.0.co;2-5 8807453

[B361] Zhao L. Longo-Guess C. Harris B. S. Lee J.-W. Ackerman S. L. (2005). Protein accumulation and neurodegeneration in the woozy mutant mouse is caused by disruption of SIL1, a cochaperone of BiP. Nat. Genet. 37, 974–979. 10.1038/ng1620 16116427

[B362] Zhao L. Rosales C. Seburn K. Ron D. Ackerman S. L. (2010). Alteration of the unfolded protein response modifies neurodegeneration in a mouse model of Marinesco-Sjögren syndrome. Hum. Mol. Genet. 19, 25–35. 10.1093/hmg/ddp464 19801575 PMC2792147

[B363] Zordan P. Croci L. Hawkes R. Consalez G. G. (2008). Comparative analysis of proneural gene expression in the embryonic cerebellum. Dev. Dyn. 237, 1726–1735. 10.1002/dvdy.21571 18498101

[B364] Zuo J. De Jager P. L. Takahashi K. A. Jiang W. Linden D. J. Heintz N. (1997). Neurodegeneration in Lurcher mice caused by mutation in delta2 glutamate receptor gene. Nature 388, 769–773. 10.1038/42009 9285588

